# Nutritional approaches in combating therapeutic resistance and enhancing treatment efficacy in cancer: the impact of ketogenic diets

**DOI:** 10.1186/s12986-025-01055-3

**Published:** 2025-12-15

**Authors:** Lei Wang, Pezhman Shafiei Asheghabadi, Saba Mashhadikhan, Sevda Nasirzade, Yeganeh Ettehad, Sepideh Gholamrezaie, Fatemeh Jafari, Maryam Rahmani, Shaghayegh Mehdizadeh, Neda Zali, Najma Farahani, Russel J. Reiter, Maliheh Entezari, Mina Alimohammadi, Afshin Taheriazam, Payman Rahimzadeh, Kiavash Hushmandi, Mehrdad Hashemi

**Affiliations:** 1 Department of Radiation Oncology, Chengdu Qingbaijiang District, People’s Hospital, Chengdu, Sichuan China; 2 Farhikhtegan Medical Convergence Sciences Research Center, Farhikhtegan Hospital, Faculty of Medicine, TeMs.C., Islamic Azad University, Tehran, Iran; 3 Basic and Molecular Epidemiology of Gastrointestinal Disorders Research Center, Research Institute for Gastroenterology and Liver Diseases, Shahid Beheshti University of Medical Sciences, Tehran, Iran; 4 Department of Biology, ET.C, Islamic Azad University, Tehran, Iran; 5 Department of Cell Systems and Anatomy, UT Health San Antonio, San Antonio, TX 78229 USA; 6 Department of Genetics, Faculty of Advanced Science and Technology, TeMs.C., Islamic Azad University Tehran, Iran; 7 Department of Immunology, School of Medicine, Shahid Beheshti University of Medical Sciences, Tehran, Iran; 8 Department of Orthopedics, Faculty of Medicine, TeMs.C., Islamic Azad University, Tehran, Iran; 9 Surgical Research Society (SRS), Students’ Scientific Research Center, Tehran University of Medical Sciences, Tehran, Iran; 10 Department of Epidemiology, University of Tehran, Tehran, Iran

**Keywords:** KD, Cancer metabolism, Therapeutic resistance, Warburg effect, Metabolic reprogramming

## Abstract

Cancer remains a major global health challenge, with therapeutic resistance significantly limiting treatment success. Traditional approaches, including surgery, chemotherapy, and radiotherapy, often encounter barriers such as metastasis and adverse effects on healthy tissues. The Warburg effect, which describes cancer cells’ reliance on glucose fermentation despite oxygen availability, has prompted investigations into alternative metabolic interventions. The ketogenic diet (KD), characterized by high fat intake and low carbohydrate intake, induces a metabolic shift that may selectively disadvantage malignant cells while preserving normal tissue function. While emerging evidence, including preclinical studies, early-phase trials, and limited clinical series, suggests that KD may help overcome treatment resistance by suppressing the PI3K/Akt/mTOR pathway, reducing insulin-like growth factor-1 (IGF-1) signaling, and enhancing cancer cell susceptibility to chemotherapy and targeted therapies, robust data from large, randomized controlled trials (RCTs) remain sparse. Most current findings derive primarily from animal models and small pilot studies, with definitive efficacy and safety in broad patient populations yet to be established. Preclinical and preliminary clinical studies indicate KD’s potential in modulating epigenetic markers, reducing inflammation, and improving patient metabolic health. However, patient adherence remains challenging and standardized protocols are still under development. This review explores the molecular mechanisms underlying KD’s anticancer effects, its role in mitigating drug resistance, and current translational insights, with emphasis on the nascent stage of high-quality clinical trial evidence and areas for future research.

## Key points

• Traditional treatments often harm healthy tissue and may trigger secondary cancers.

• The Ketogenic diet (KD) targets cancer metabolism, potentially sparing healthy cells.

• Combining KDs with standard therapies may help overcome drug resistance.

**Graphical abstract:**

## Graphical abstract

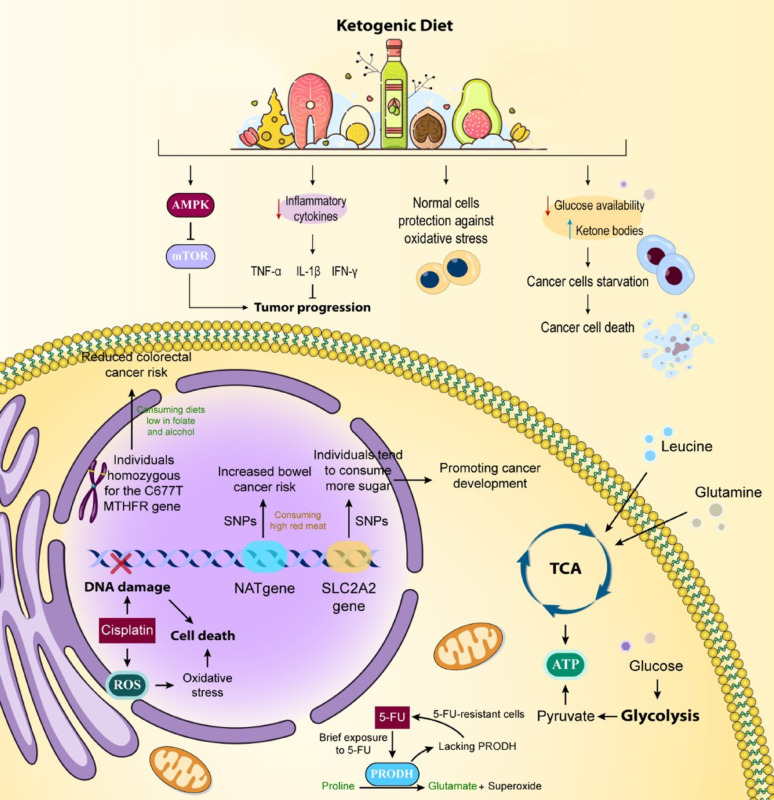

## Introduction

Cancer continues to be one of the most significant health challenges of our time, emerging as a leading cause of death worldwide, particularly as treatment resistance grows and incident rates increase [1]. Statistical data from 2020 reveals concerning figures in the United States: over 1.8 million new cancer diagnoses and approximately 606,000 deaths, with young people accounting for 89,500 cases and 9,270 fatalities annually [2]. While traditional treatment methods such as surgical procedures, chemotherapeutic drugs, radiotherapy, and targeted therapies like monoclonal antibodies and tyrosine kinase inhibitors have shown success in tumor control and elimination, they face significant limitations [3]. A breakthrough in understanding cancer cell metabolism came in the 1920 s when Otto Warburg identified the phenomenon now known as the Warburg effect [4]. This discovery revealed that cancer cells, unlike normal cells, predominantly rely on fermentation for energy production, even in oxygen-rich conditions, due to compromised mitochondrial function [5]. This metabolic adaptation supports the rapid production of building blocks necessary for cell division [6]. Based on this understanding, scientists have begun exploring nutritional approaches, particularly the ketogenic diets (KDs) and intermittent fasting protocols, as complementary therapeutic strategies that target cancer’s unique metabolic requirements [7–9].

A KDary approach, which consists of predominantly fats (80–90%), sufficient protein, and severely restricted carbohydrates (2–8%), promotes a metabolic transition from glucose-dependent energy production to mitochondrial fat oxidation [10]. This metabolic shift shows promise in cancer management by targeting malignant cells while preserving healthy tissue function. Research has shown that this dietary intervention reduces insulin-like growth factor-1 (IGF-1) and increases ketone body production, with studies in animal models demonstrating that limiting carbohydrate intake can impede cancer progression [11, 12].

Recent research indicates that KDs might help address the challenge of drug resistance in cancer treatment through various molecular mechanisms [13]. By restricting glucose availability and forcing metabolic adaptation, this dietary approach could potentially resensitize resistant cancer cells to chemotherapy agents [13]. Additionally, the diet’s suppressive effect on the PI3K/Akt/mTOR pathway might enhance the effectiveness of targeted therapies [14]. The diet-induced reduction in insulin and IGF-1 signaling could also help counteract survival pathways that typically contribute to treatment resistance [15]. While these mechanisms suggest promising potential for KDs in overcoming therapeutic resistance, definitive clinical evidence is still being gathered through ongoing research. This comprehensive review explores the KD’s development over time, examines cancer metabolism through Warburg’s pioneering insights, details the diet’s anti-cancer mechanisms, and evaluates both laboratory and clinical evidence supporting its use alongside conventional cancer treatments.

## Therapeutic resistance in cancer: mechanisms and implications

### Overview of therapeutic resistance

Cancer cells exhibiting drug resistance display distinctive epigenetic markers on their DNA, histones, and various regulatory elements involved in gene expression; research has identified specific epigenetic modifications, such as DNA hypermethylation patterns associated with tumor suppressor gene inactivation, as crucial indicators of cancer development [16, 17]. An illustrative example is the demethylation of the ABCB1 transporter gene, which reduces chemotherapy drug retention within cancer cells, resulting in multiple drug resistance [18]. Additionally, epigenetic modifications can compromise DNA repair mechanisms, as seen in the hypermethylation of hMLH1, contributing to colorectal cancer progression and treatment resistance [19].

The Food and Drug Administration (FDA) has authorized two categories of epigenetic-modifying medications: DNA methylation inhibitors (including 5-azacitidine and Decitabine) and histone deacetylase inhibitors (such as Vorinostat, Belinostat, Romidepsin, and Panobinostat) [20, 21]. While Decitabine may not directly inhibit tumor growth, its ability to block DNA methylation enhances tumor sensitivity to various chemotherapy agents, including carboplatin, cisplatin, and 5-FU [22]. In colorectal cancers, specific epigenetic signatures have been identified; early detection markers include methylation patterns in MDF1, SSTR2, and CMTM3, while CLDN11 hypermethylation correlates with metastasis and poor outcomes [23, 24]. TUSC3 silencing through promoter hypermethylation increases EGFR expression, promoting cancer cell survival [25]. Novel therapeutic approaches may include DNA methyltransferase inhibitors and histone deacetylase targeting drugs; recent research highlights the potential of new compounds like CUDC-101 as promising treatments for solid tumors [26]. MicroRNAs (miRNAs), short non-coding sequences of 19–25 nucleotides, regulate gene expression through post-transcriptional modifications. These epigenetic regulators significantly influence chemotherapy resistance development in various cancers [27]. Studies have revealed their impact on drug resistance genes, cell proliferation, cell cycle regulation, and apoptosis pathways, suggesting their potential as predictive markers for treatment response [27]. The catalog of cancer-associated miRNAs continues to expand.

Post-translational histone modifications (PTMs) are recognized by specialized protein domains: chromo-domains bind methylation, bromo-domains detect acetylation, BCRT domains recognize phosphorylation, and PHD domains interact with methylation marks. Chromatin remodeling complexes like SWI/SNF, ISWI, CHD, and INO80 further modulate chromatin structure. This interplay between DNA and histone modifications generates extensive non-genetic diversity, enabling precise biological control. Genomic analyses reveal frequent mutations in enzymes responsible for these modifications and remodeling factors, contributing to cancer development [28].

The establishment of tumor-specific epigenetic patterns not only triggers cancer formation but also enables the accumulation of additional genetic and epigenetic alterations, particularly those that enhance tolerance against therapeutic agents [29]. Introducing drug-free intervals may restore treatment sensitivity by allowing epigenetic resistance markers to revert. During these periods, non-genetically resistant cancer cells can be targeted using a combination of conventional treatments and epigenetic-modifying agents, such as HDACi, DNMTi, EPZ004777, and BET-I. This approach can transform drug-resistant epigenetic profiles back to drug-sensitive states, although genetically resistant cells may persist. A therapeutic strategy combining conventional and epigenetic drugs may effectively reduce tumor size while preventing the development of genetic resistance to targeted therapies [29, 30]. Cancer cells develop resistance to chemotherapy through various mechanisms, including enhanced drug efflux, reduced drug uptake, drug deactivation, modifications to drug targets, and resistance to cell death. Scientists are still uncovering the complex processes involved in how cancer cells transport chemotherapy drugs in and out of cells [31, 32]. One notable example involves the reduced folate carrier (RFC), which facilitates the uptake of drugs like methotrexate (MTX) and thymidylate synthase inhibitors. Research has shown that tumors with mutations affecting RFC function can become resistant to MTX. The impact of RFC on treatment outcomes is demonstrated in childhood acute lymphoblastic leukemia (ALL), where patients with the RFC genotype (80AA) show elevated plasma MTX concentrations, suggesting compromised drug uptake and increased mortality rates [33].

Cancer cells can resist treatment by circumventing programmed cell death mechanisms. Caspases, which are crucial enzymes in apoptosis, function by breaking down cellular components and activating secondary death pathways. The Bcl-2 protein family, comprising both pro-death and anti-death members, plays a vital role in cell survival decisions. In many cancers, anti-death proteins become overactive, enabling cells to survive chemotherapy exposure. Treatment resistance often stems from either increased anti-death signals or decreased pro-death protein function [34–37].

The translationally controlled tumor protein (TCTP) helps cancer cells survive by disrupting the Apaf-1 complex, thereby preventing caspase activation. High levels of TCTP often correlate with chemotherapy resistance due to its interference with cell death mechanisms, particularly in HeLa cells [38]. Another factor in treatment resistance involves the inhibitors of apoptosis proteins (IAPs), particularly X-linked IAP (XIAP), which shows the strongest anti-death effects through its ability to block caspase-9 activation [39]. Dysregulation of IAP proteins and NF-κB signaling in cancers contributes significantly to treatment resistance and poor outcomes, particularly in acute myeloid leukemia [40]. XIAP deficiency has been linked to X-linked lymphoproliferative syndrome type 2, suggesting that targeting IAPs with small-molecule inhibitors could potentially enhance cancer cell death [41].

In colon cancer cells, impaired trafficking of death receptors TRAIL-R1 and TRAIL-R2 from the endoplasmic reticulum to the cell surface creates resistance to TRAIL-induced death [42]. Numerous cancers exhibit dysfunction of TRAIL receptors, and alterations in decoy receptors provide another mechanism for evading cell death. For instance, gastric cancers often show elevated levels of the decoy receptor TRAIL-R3 [43]. Additionally, c-FLIP, a key anti-apoptotic regulator, can block caspase-8 recruitment and suppress chemotherapy-induced TRAIL-mediated cell death [44].

**Fig. 1:**
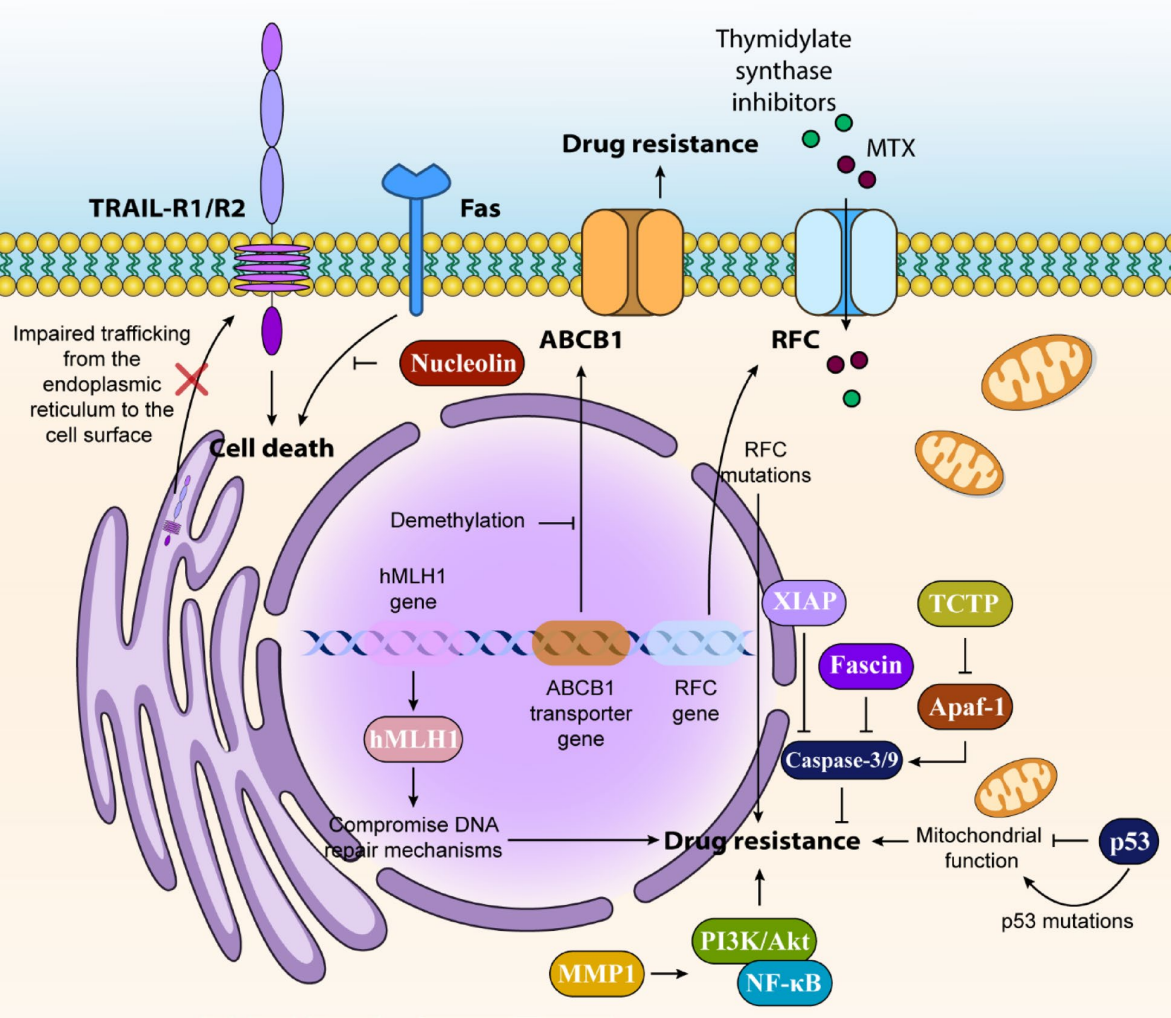
Mechanisms and Implications of Cancer Therapeutic Resistance

Various signaling molecules influence cell death pathways and alter cancer cells’ response to chemotherapy. The JNK and p38-MAPK pathways regulate multiple Bcl-2 family proteins, while JNK can activate PUMA and promote cell death in resistant cells through Akt/Fox03a signaling [45]. In breast cancer, the protein fascin promotes treatment resistance by increasing anti-apoptotic protein expression while suppressing pro-apoptotic proteins like caspases-3 and − 9 [46] (Fig. 1).

### Metabolic reprogramming in cancer and its role in resistance

**Fig. 2:**
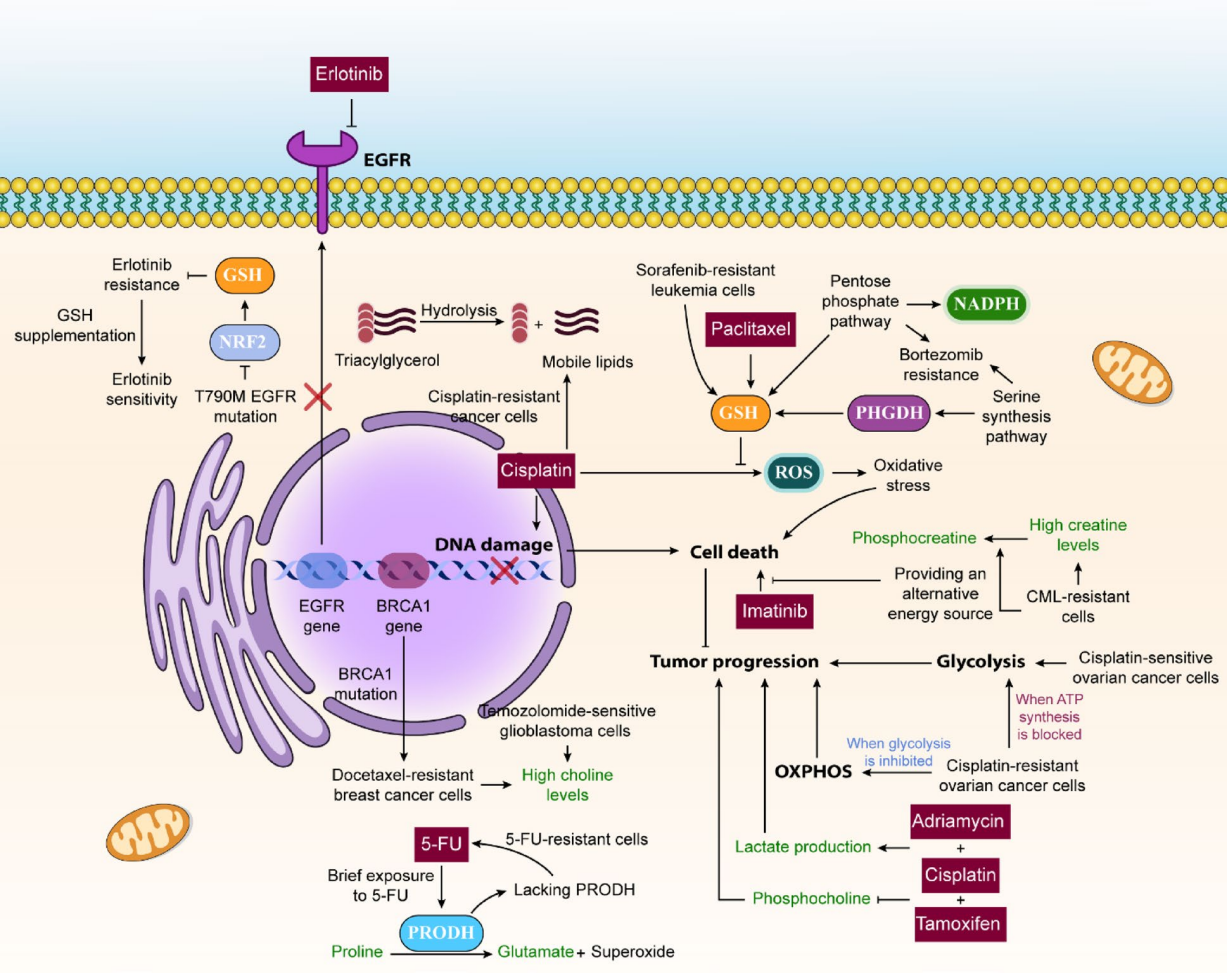
A Schematic Representation of the Role of Metabolic Reprogramming in Cancer Drug Resistance

Through metabolomic analysis, researchers have identified several key metabolic adaptations in resistant tumor cells responding to various chemotherapy treatments **(**Fig. 2**)**. Cell survival and growth depend heavily on maintaining redox balance and antioxidant capabilities. Platinum-based drugs like cisplatin, widely used in cancer treatment, such as ovarian cancer, work by triggering cell death through DNA damage and oxidative stress from ROS [47–49]. Research shows that cisplatin-resistant lung cancer cells undergo metabolic reprogramming, leading to increased ROS production and greater reliance on oxidative metabolism rather than glycolysis. The elevated ROS levels and metabolic changes promote epithelial-mesenchymal transition (EMT) [47, 50, 51].

These resistant cells show increased glutamine consumption and vulnerability to glutamine restriction. They convert glutamine to glutamate for GSH synthesis, which helps reduce cellular ROS levels. Targeting glutamate metabolism specifically eliminates cisplatin-resistant cells [47]. While glutamine provides the glutamate and glycine components of GSH, cysteine comes from different sources. Cysteine, which provides GSH’s thiol group, requires both cellular uptake and limited internal production to maintain GSH availability. The thiol components from GSH, cysteine-glycine, and cysteine can neutralize platinum molecules, preventing them from damaging cellular components [52].

Cysteine plays a crucial role in cancer cell growth, survival, and metabolic adaptation. It influences how cancer cells, particularly ovarian cancer cells, adapt to low oxygen conditions, contributing to resistance against platinum-based chemotherapies. Controlling cysteine availability could potentially reverse both hypoxia and carboplatin resistance. Additionally, cysteine serves as an alternative pyruvate source for cancer cells, with its breakdown providing approximately 20% of cellular pyruvate [53].

Interestingly, in the case of erlotinib (an EGFR inhibitor), T790M EGFR mutation-induced resistance shows a different pattern. These resistant cells exhibit reduced GSH levels due to T790M mutation’s suppression of NRF2 activity, which controls GSH-synthesizing enzyme expression [54]. GSH supplementation restored erlotinib sensitivity in resistant cells, while GSH reduction in sensitive cells induced resistance. This unusual relationship between decreased GSH levels and EGFR tyrosine kinase inhibitor resistance warrants further investigation [55].

Research has demonstrated that bortezomib-resistant cells in multiple myeloma redirect glucose toward the pentose phosphate pathway and serine synthesis pathway, enhancing their antioxidant capabilities. The PPP generates NADPH, helping maintain GSH levels and cellular redox equilibrium. Additionally, elevated PHGDH activity in the serine synthesis pathway contributes to increased GSH production, promoting cell survival and bortezomib resistance [56, 57].

Regarding lipid metabolism adaptation, studies indicate significant metabolic reprogramming in chemotherapy-treated cancer cells, as lipid metabolism changes were observed in cisplatin-resistant bladder cancer through lipidomic analysis. Bladder cancer cells resistant to cisplatin showed distinct metabolites, primarily involving phospholipids, fatty acids, amino acids, and energy metabolism components [58].

Higher choline levels were detected in docetaxel-resistant BRCA1-mutated mammary tumors compared to sensitive ones [59]. In contrast, temozolomide-sensitive glioblastoma cells showed elevated choline and phosphorylcholine levels compared to resistant cells [60]. Generally, increased phospholipid levels correlate with drug resistance, though exceptions exist.

Cancer cells exhibit distinctive energy metabolism patterns as a fundamental characteristic, employing bioenergetic adaptations to survive drug treatments. Studies show that while cisplatin-sensitive ovarian cancer cells primarily utilize glycolysis, their resistant variants demonstrate metabolic flexibility. These resistant cells can either increase glycolytic activity when ATP synthesis is blocked or enhance oxidative phosphorylation (OXPHOS) when glycolysis is inhibited, showing their ability to alternate between these energy pathways [61].

Regarding glucose metabolism, cancer cells typically favor aerobic glycolysis. Research on sorafenib-resistant leukemia cells revealed increased glucose requirements and reduced pentose phosphate pathway (PPP) activity [62]. Studies on imatinib-resistant CML cells have yielded conflicting results. Initial research indicated increased glucose uptake and glycolysis in resistant BCR-ABL-positive cells during imatinib treatment, while later studies found decreased glucose consumption and lactate production [63]. The reason for these contradictory findings remains unclear. Studies of flavopiridol-resistant prostate cancer cells also demonstrated enhanced glycolysis and reduced sensitivity to cisplatin and docetaxel [64].

In terms of polyamine metabolism, these compounds play vital roles in eukaryotic cell growth. Significant increases in polyamine synthesis were observed in platinum-resistant ovarian cancer cells [65]. Research on colorectal cancer revealed that tumor-associated macrophages (TAMs) release putrescine when exposed to 5-Fluorouracil (5-FU), contributing to treatment resistance. Researchers found that inhibiting ornithine decarboxylase, crucial for putrescine production, reduced resistance to 5-FU and enhanced its anti-tumor effects [66]. The progressive shift of adriamycin-sensitive cells toward metabolic patterns similar to resistant cells provides evidence for metabolic adaptation during chemoresistance development [67].

Metabolic adaptations to chemotherapy appear to be dynamic processes. For instance, brief exposure of gastric cancer cells to 5-FU results in decreased proline and elevated glutamate levels, potentially linked to increased proline dehydrogenase (PRODH) activity. However, 5-FU-resistant cells show minimal changes in these metabolites, lacking PRODH upregulation after treatment. PRODH, which converts proline to glutamate while generating superoxide, becomes more active when 5-FU disrupts nucleotide synthesis. This process, involving mitochondrial superoxide production, contributes to resistance development [68].

Furthermore, combination therapies can produce unique metabolic effects. In breast cancer cells, combining cisplatin with tamoxifen decreases phosphocholine levels, while cisplatin plus adriamycin increases lactate production [69]. Although precisely predicting these metabolic changes remains challenging, researchers are working to identify plasma biomarkers that could indicate drug resistance in human epithelial ovarian cancer (Table 1) [70].

**Table 1:** The metabolic adaptations of drug-resistant cancer cells

Metabolic adaptation	Key findings	Drug involved	Cancer type	References
Oxidative Stress Adaptation	Increased glutathione (GSH) synthesis to cope with oxidative stress.	Cisplatin	Ovarian, Lung Cancer	[47, 51, 71]
	Cysteine uptake critical for GSH bioavailability and platinum resistance.	Carboplatin	Ovarian cancer	[53, 72, 73]
	Increased GSH levels in paclitaxel-resistant cells.	Paclitaxel (Taxol)	Triple Negative Breast Cancer	[74]
	Downregulated GSH levels in erlotinib-resistant EGFR-mutated cells.	Erlotinib	EGFR-driven Cancers	[54]
Lipid Metabolism Adaptation	Higher basal lipid content in cisplatin-resistant cells.	Cisplatin	Ovarian and Bladder Cancers	[58, 71]
	Altered choline phospholipid metabolism in erlotinib-resistant cells.	Erlotinib	Pancreatic Cancer (HPAC cells)	[75]
	Increased choline levels in docetaxel-resistant BRCA1-mutated tumors.	Docetaxel	Breast Cancer	[59]
	Upregulation of choline in temozolomide-sensitive GBM cells.	Temozolomide	Glioblastoma (GBM)	[60]
Bioenergetic Adaptation	Switch between glycolysis and oxidative phosphorylation (OXPHOS).	Cisplatin	Ovarian Cancer	[61]
	Elevated creatine and phosphocreatine levels in imatinib-resistant cells.	Imatinib	Chronic Myelogenous Leukemia	[76]
Dependence on Glucose/Glycolysis	Increased glucose demand and reduced PPP flux in sorafenib-resistant cells.	Sorafenib	Leukemia	[77]
	Contradictory glycolysis changes in imatinib-resistant CML cells.	Imatinib	Chronic Myelogenous Leukemia	[76, 78]
	Enhanced glycolysis in flavopiridol-resistant prostate cancer cells.	Flavopiridol	Prostate Cancer	[64]
Polyamine Synthesis	Increased polyamine synthesis in platinum-resistant cells.	Platinum	Ovarian Cancer	[49]
	Putrescine secretion by TAMs induces 5-FU resistance.	5-Fluorouracil (5-FU)	Colorectal Cancer	[66]

## Warburg effect and cancer metabolism

The metabolic differences between cancer cells and normal tissue cells represent a fundamental characteristic of cancer. These extensive metabolic modifications are primarily triggered by oncogenic signaling pathways and modified metabolic enzymes, enabling cancer cells to meet their growth requirements in environments with variable nutrient availability. However, this adaptation creates a dependency on continuous nutrient and energy supplies, beyond the well-documented glucose metabolism alterations, leading to enhanced production of amino acids and fatty acids to support tumor growth [79, 80].

**Fig. 3:**
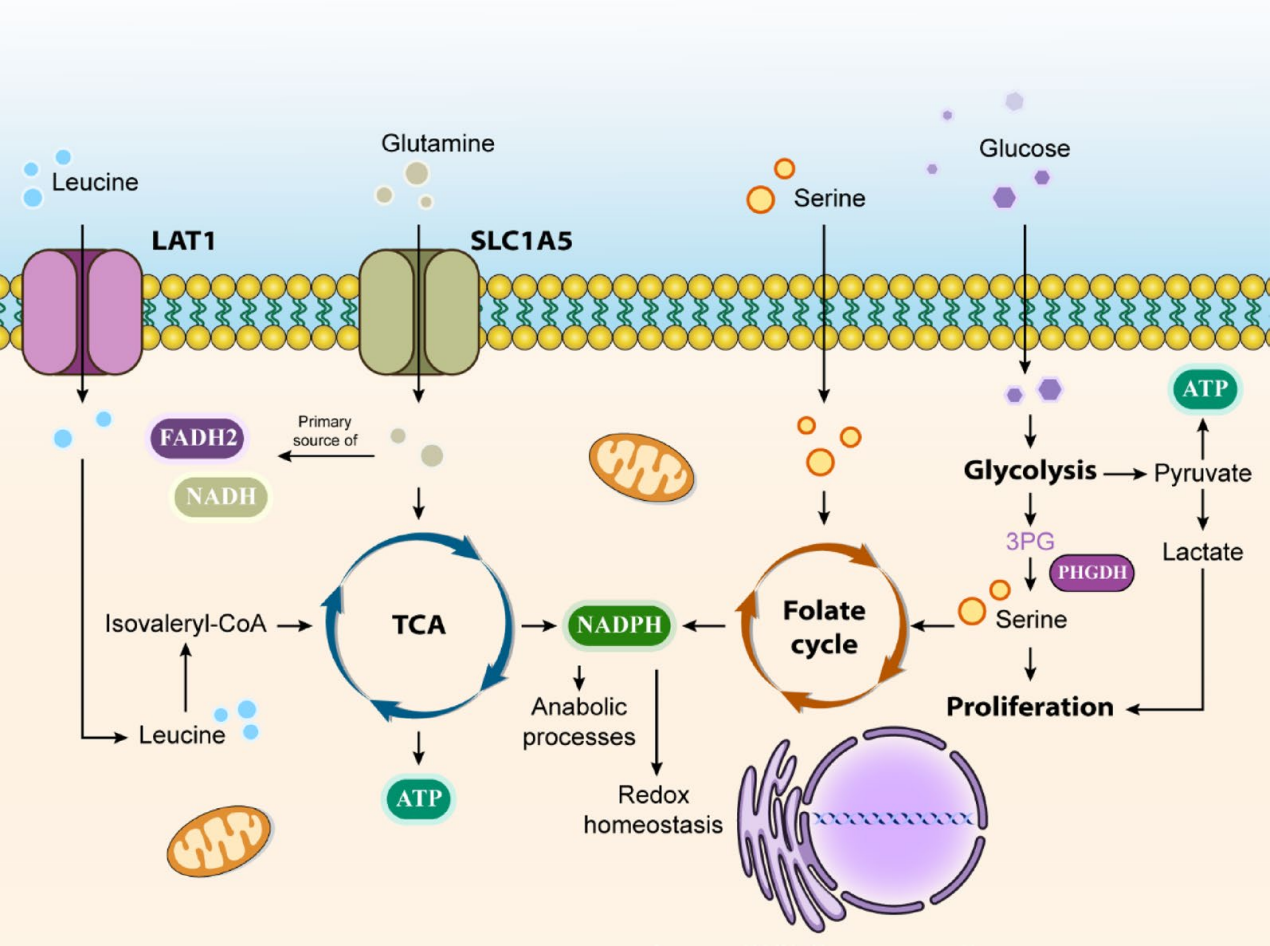
Cancer Metabolism: The Crosstalk between Metabolism of Organic Macromolecules and Cancer Progression

Cancer cells characteristically demonstrate elevated glucose consumption to generate intermediates essential for synthesizing lipids, proteins, and nucleic acids. They also maintain TCA cycle intermediate levels through increased glutamine uptake and metabolism. This heightened biosynthetic activity necessitates greater NADPH production, which supports both anabolic processes and cellular redox homeostasis. The transformation of cells during cancer development involves epigenetic changes dependent on various metabolites: acetyl-CoA (acetylation), NAD (deacetylation), SAM (methylation), α-ketoglutarate (demethylation), and UDP-GlcNAc (glycosylation). Modern cancer research has revealed that understanding genetic mutations alone is insufficient; cancer cells exist within complex tumor tissues, interacting with their microenvironment and developing characteristics that enhance their survival, growth, and spread [81–84] (Fig. 3).

Regarding glutamine metabolism, this most abundant plasma amino acid serves crucial roles in cancer cell proliferation. It provides nitrogen for nucleotide, amino acid, and hexosamine synthesis, while contributing both nitrogen and carbon to various cellular reactions. Glutamine is particularly vital as it acts as a precursor for non-essential amino acids (NEAAs) and fatty acid synthesis [85, 86].

The non-essential amino acid serine plays vital roles in cellular proliferation and survival. Cells can either synthesize serine from glycolysis-derived 3-phosphoglyceric acid (3PG) or acquire it from external sources [87]. As the third most utilized metabolite by cancer cells, after glucose and glutamine, serine provides one-carbon units to the folate cycle, supporting nucleotide synthesis, methylation processes, and NADPH generation for antioxidant defense [88]. Given cancer cells’ strong dependence on serine, therapeutic strategies targeting either de novo serine synthesis or external serine availability show promise. This has led to increased interest in phosphoglycerate dehydrogenase (PHGDH), a key enzyme in serine synthesis, as a potential therapeutic target [89].

Leucine, an essential branched-chain amino acid (BCAA), must be obtained through protein-rich foods such as meat, dairy, and legumes. This amino acid serves crucial functions in protein synthesis and various metabolic processes, including blood sugar regulation, muscle and bone tissue maintenance, and growth hormone production. As a ketogenic amino acid, leucine breaks down into ketone bodies - acetoacetate and β-hydroxybutyrate. Through its activation of mTOR and S6K1, leucine can interfere with insulin signaling, resulting in decreased glucose utilization in skeletal muscle tissue [90–92].

Leucine serves as an energy source when enzymatically converted to isovaleryl-CoA and utilized in the TCA cycle for ATP production. During tumor progression, cancer cells require alternative energy sources to sustain their rapid growth. Studies have documented elevated BCAA levels, including leucine, in the plasma of patients with pancreatic cancer and melanoma. Research indicates that leucine activates mTORC1 by providing acetyl-CoA to the EP300 acetyltransferase, which in turn influences Raptor acetylation at K1097. Many cancers rely on mTOR activity to maintain cellular growth and division [93–95].

Research indicates that limiting leucine availability may inhibit cell growth, promote apoptosis, and reduce FASN expression in breast cancer. The L-type amino acid transporter (LAT1) facilitates cellular leucine uptake; therefore, LAT1 inhibition leads to reduced mTOR signaling and tumor growth suppression. However, research also demonstrates the benefits of leucine-rich diets for cancer patients experiencing malnutrition and cachexia [96, 97].

## KDs: composition and biological impact

The KD, high in fat and low in protein and carbohydrates, shows therapeutic potential as a safe, cost-effective, and easy-to-implement strategy in cancer treatment. It can be used alone or alongside conventional therapies to enhance chemotherapy efficacy, protect healthy tissue, reduce inflammation, and modulate proteins and factors like MMPs, HDACs, AMPK, PK, and P53. Evidence from human and animal studies supports KD’s role in reducing tumor growth and improving patient outcomes [98]. Malignant cells exhibit altered metabolism, heavily relying on glucose via the Warburg effect and producing lactate even in oxygen-poor conditions. This dependence makes them vulnerable to the KD, which lowers glucose and raises ketone bodies that cancer cells cannot utilize, effectively starving them. In contrast, healthy cells adapt by metabolizing fatty acids into ketones for efficient energy production, preserving normal function [99]. It should be considered that if KD were a low protein diet, limitations would occur in the context of malnutrition and sarcopenia. Regarding the heterogeneity of KD protocols, Table 2 provides details of varied KD composition according to clinical studies and RCTs.

**Table 2:** Ketogenic diet composition in cancer clinical studies

Cancer type	Fat %	Protein %	Carb %	Ketogenic ratio (Fat: Protein + Carb)	Notes	Reference
Ovarian/Endometrial Cancer	70	25	5	~ 2.8:1	Protein notably higher than theory, carbs restricted	[100]
Breast Cancer	80–85	16–18	2–4	1.6:1–2:1	Closer to classical KD but with increased protein	[101]
Pancreatobiliary Cancer	80	15–20	3–6	1.75:1	Moderate protein	[102]
Breast Cancer	55	19	6	~ 1.4:1	Use of MCT; higher protein, lower fat than standard KD	[103]
Pancreatobiliary Cancer post-pancreatectomy	70–80	15–25	3–6	1.05:1–1.75:1	Moderate protein range	[104]

### KD and Cancer-related mechanisms, genes, and/or proteins

Inflammation is a complex response in vascular tissues after injury or during chronic disease, involving cytokines, chemokines, and transcription factors, and is closely linked to cancer. In some cancers, inflammation precedes malignancy, while in others it promotes tumor growth, spread, and therapy resistance. Studies show that KD reduces inflammatory cytokines (TNF-α, IL-1β, IFN-γ), potentially improving cancer treatment and prevention [105]. TNF-α can trigger IL-8 release and activate NF-kB transcription factors, crucial elements in cancer progression [106].

Research focusing on GBM has revealed that combining KD with DON (a glutamine antagonist) significantly reduces tumor growth, enhances survival rates, and decreases inflammation [107]. In breast cancer, TNF-α serves multiple functions in disease progression and metastasis. A randomized control trial by Khodabakhshi et al. showed significant TNF-α reduction in breast cancer patients after 12 weeks of KD, attributed to MMP-9 suppression and PPARγ activation [103].

Cyclooxygenase (COX), existing in two isoforms (COX-1 and COX-2), plays a crucial role in prostaglandin and eicosanoid production from arachidonic acid. COX-2 overexpression has been documented in various cancers, including colon, breast, and lung cancers. Given COX-2’s significant influence on tumor development stages, selective COX-2 inhibitors show promise in cancer prevention and treatment. In vitro studies using mouse glioma models have shown that KD significantly reduces COX-2 expression, while combination studies with radiation therapy suggest decreased expression of COX-2 and other inflammatory markers. However, additional research is needed to definitively establish KD’s effectiveness in COX-2 expression modulation [108, 109].

Matrix metalloproteinases (MMPs), which function as zinc-dependent endopeptidases, play an essential role in breaking down the extracellular matrix (ECM). The ECM is vital for cellular cohesion and maintaining bodily structure. Beyond ECM degradation, these enzymes significantly influence various aspects of cancer development, including its spread, invasion, and blood vessel formation. Research has identified elevated levels of several MMPs across multiple cancer types, including colorectal cancer, glioblastoma, and gastric cancer. A notable member, MMP-9, belongs to the gelatinase subfamily and originates from inflammatory cells, tumor-adjacent stromal cells, or the cancer cells themselves. Research suggests that implementing a KD alongside traditional cancer treatments may significantly inhibit MMP-9 expression in mouse models of colon cancer [110–113].

HDACs represent a crucial protein family that governs gene transcription and protein function by removing acetyl groups from specific lysine residues in core histone proteins bound to DNA. These enzymes are fundamental in regulating cellular processes closely associated with cancer development and progression. HDAC inhibitors have demonstrated the ability to modify gene expression patterns and influence various cellular processes, including growth inhibition, differentiation, cell death, and apoptosis activation. While multiple correlative studies have demonstrated connections between KDs, ketone bodies, and HDAC suppression in human cancers, this area requires further investigation [114–116].

Research findings have demonstrated that KDs play a significant role in suppressing mutant p53 variants through β-hydroxybutyrylation and deacetylation processes and promoting cellular death. This decrease in mutant p53 levels contributes to extended survival. Additionally, glucose-restricted dietary approaches facilitate p53 mutant deacetylation and breakdown; consequently, KDs either inhibit p53 mutant function or suppress gene expression throughout cancer development and advancement [117].

The AMP-activated protein kinase (AMPK) represents a serine/threonine kinase enzyme present across multiple cell types. AMPK modulation presents a promising therapeutic approach for treating diverse malignancies, including colorectal, pulmonary, and hepatic cancers. AMPK stimulation correlates with tumor suppressor pathways involving p53 and LKB1, cellular proliferation inhibition, inflammatory response reduction, growth restriction, and cell cycle disruption; therefore, AMPK serves a critical function in cancer prevention. Energy shortage conditions, such as glucose limitation and oxygen deprivation, trigger AMPK activation. Furthermore, pharmaceutical agents, including metformin, curcumin, quercetin, and certain anti-inflammatory medications, can stimulate AMPK activity. The metabolic shift from glucose utilization to ketone body consumption in malignant cells following KD implementation has been associated with enhanced AMPK stimulation [118–122].

miRNAs are known for their ability to modulate gene expression through target mRNA binding to control protein production or mRNA breakdown. miRNAs can influence tumors at every stage, encompassing initiation, advancement, apoptosis, blood vessel formation, proliferation, and cellular differentiation [123].

In this context, miR-21 regulates numerous biological mechanisms, including inflammatory responses, fibrotic processes, and carcinogenesis. Specifically, it has been found to regulate transforming growth factor β (TGF-β) through SMAD signaling networks in renal fibrosis and inflammation. Additionally, miR-21 facilitates colorectal cancer progression by targeting tumor suppressor messenger RNAs, including tropomyosin 1, programmed cell death 4 (PDCD4), and phosphatase and tensin homolog (PTEN) [124, 125].

Alterations in miRNAs, including Let-7, miR-21, and miR-145, impact colorectal cancer mechanisms. Let-7 miRNA acts as a tumor suppressor by modulating Ras, c-myc, and p53, essential for colorectal cancer initiation and progression. Oncogenic miR-21 is upregulated in tumors and targets tumor suppressors such as PTEN and PDCD1. The role of miR-145 is controversial; while previously seen as tumor-suppressive, recent studies link its upregulation to enhanced cancer cell migration and invasion. Environmental and lifestyle factors, including diet, influence miRNA expression in colorectal cancer [126–129].

Additional comprehensive mechanistic studies are required to establish the potential relationship between miRNA function and KD implementation in cancer development (Table 3).

**Table 3:** The relationship between inflammation, cancer, and the KD

Topic	Key findings	Mechanism/Effect	References
TNF-α & Cancer	KD/DON combination reduces TNF-α expression in GBM	Glutamine antagonism	[130]
	KD suppresses TNF-α in breast cancer via MMP-9 and PPARγ	PPARγ activation	[131]
MMPs & Cancer	KD reduces MMP-9 expression in cancer	Anti-metastatic effects	[113]
PKM2 & Cancer	KD attenuates PKM2, reducing glucose uptake and lactate production	Metabolic reprogramming	[13]
	Ketone bodies (particularly BHB) inhibit PKM2, reducing ATP and promoting apoptosis	Glycolysis inhibition	[132]
p53 & Cancer	KD (p53 β-hydroxybutyrylation) induces deacetylation and degradation of mutant p53	Epigenetic regulation	[117]
Epigenetics & Cancer	Ketone bodies (BHB) modulate DNA methylation and histone modifications.	β-hydroxybutyrylation	[133, 134]

Numerous studies support KDs or high-fat, low-carbohydrate, adequate-protein nutrition as effective cancer therapies or preventive measures, alone or combined with drugs. These dietary approaches influence pathways like insulin signaling, PI3K, AKT, mTOR, ketone metabolism, adiponectin, leptin, and IGF-1. Both preclinical and clinical research confirm KD’s anti-aging and anticancer benefits. Combined with physical activity, KD reduces cancer risk across multiple malignancies by modulating metabolism and molecular signaling [135].

High-fat diets, especially those rich in saturated fats, can induce inflammation by mimicking lipopolysaccharide (LPS) actions, triggering inflammatory responses via receptors on macrophages and innate immune cells. In contrast, polyunsaturated fats like omega-3 fatty acids (EPA, DHA) exhibit anti-inflammatory effects. Chronic inflammation, characterized by elevated cytokines and nuclear factor kappaB (NF-κB) signaling, is a cancer hallmark. Low-carbohydrate, high-fat diets emphasizing unsaturated fats reduce tumor-associated macrophages, cytokines, NF-κB, and COX-2 expression, providing cancer prevention benefits. KD metabolism produces ketone bodies, with BHB activating mitochondrial uncoupling protein-2 (UCP-2), helping modulate inflammation and metabolism [136]. Conversely, its deficiency may cause excessive ROS, pro-inflammatory cytokine release, and sustained NF-κB activation. Consequently, ketone bodies serve essential functions in reducing oxidative stress and prolonging patient survival. Adiponectin and leptin represent peptide hormones synthesized by visceral white adipose tissue. Adiponectin demonstrates inverse correlation with leptin and other adipokines. Reduced adiponectin levels have been associated with type 2 diabetes, insulin resistance, metabolic syndrome, hypertension, cardiovascular disease, and cancer [137].

**Fig. 4:**
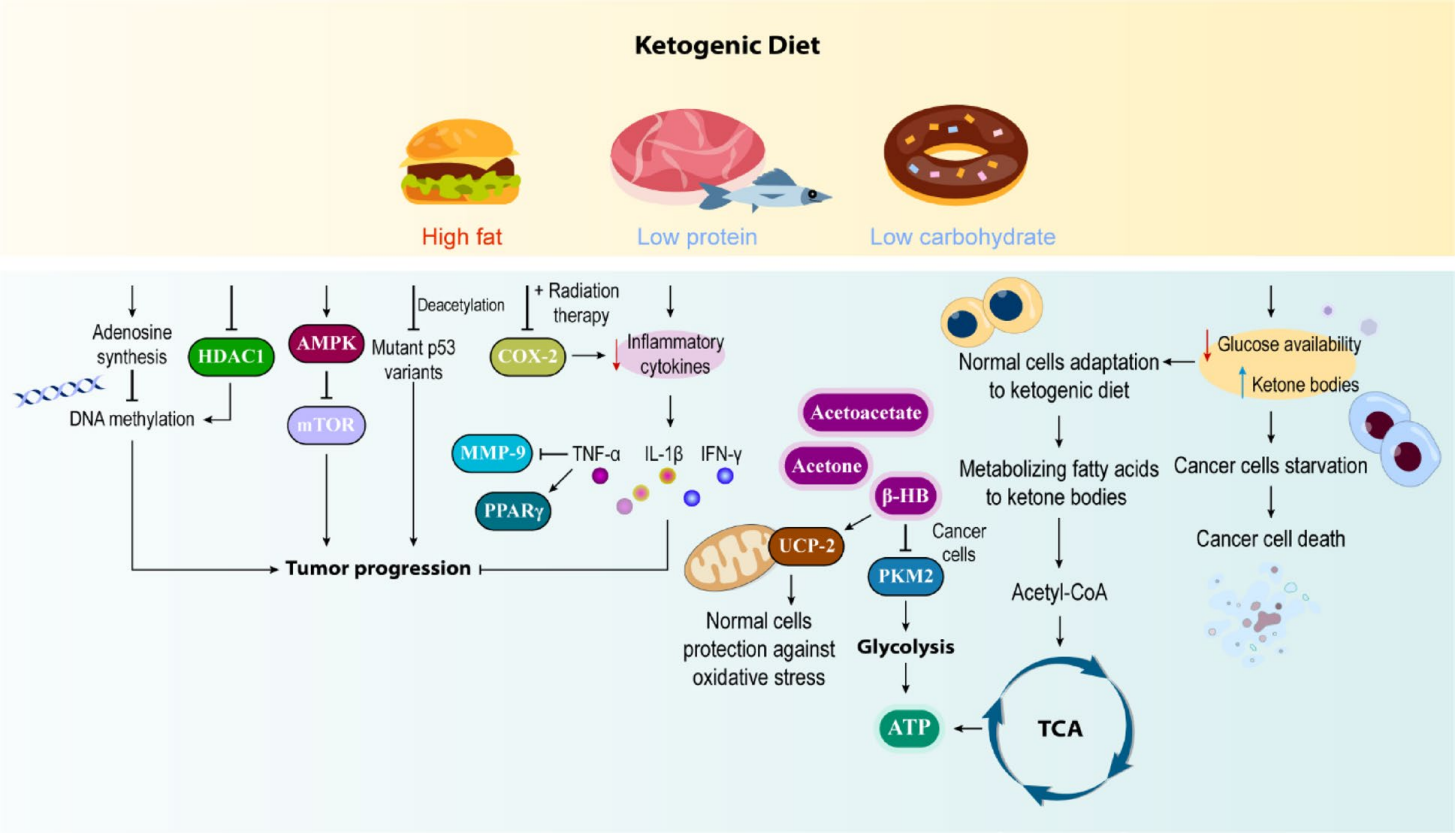
KDs and Cancer-related Molecular Mechanisms: Mechanistic Overview

Finally, a direct relationship has been established between high-calorie diets and cancer risk, along with cancer prevention approaches through lifestyle modifications, including physical activity, exercise, and healthy diets rich in fruits, vegetables, and whole grains, while limiting red meat and saturated fat consumption. KDs may not completely prevent tumor development, but can delay tumorigenesis and enhance survival rates. Furthermore, KD demonstrates synergistic benefits for cancer treatment when combined with chemotherapy or alternative cancer therapies (Fig. 4).

## KDs and their role in overcoming therapeutic resistance

KDs are emerging as metabolic interventions to combat therapeutic resistance across multiple diseases. By shifting the body’s primary energy source from glucose to ketones, KDs disrupt pathological metabolic pathways and enhance treatment efficacy in drug-resistant conditions. Pre-clinical studies demonstrate that KDs sensitize tumors to chemotherapy and targeted therapies by exploiting metabolic vulnerabilities. In PI3K inhibitor-resistant cancer models, KDs reduced glucose availability to tumors, overcoming drug resistance and inhibiting growth. A meta-analysis of animal studies showed KD significantly extended survival time and reduced tumor weight [138]. While compliance remains challenging, emerging evidence positions KDs as adjuvant therapies to overcome resistance in oncology, as some examples have been discussed in the following.

Resistance to immune checkpoint blockade (ICB) therapy is a major issue in prostate cancer due to its immunosuppressive microenvironment. Recent research by Murphy et al. (2024) shows that the KD and its metabolite BHB may enhance ICB effectiveness in preclinical, ICB-resistant prostate cancer models. Their study tested combinations of anti-PD1 and anti-CTLA4 antibodies with vorinostat, a cyclic KD, or BHB supplementation using resistant PCa cell lines [139]. Cyclic KD (CKD) and BHB supplementation both delayed tumor growth as monotherapies in resistant prostate cancer models. CKD’s anti-tumor effects relied on BHB and a functional adaptive immune system. Combining ICB with HDAC inhibition, CKD, or BHB enhanced tumor control synergistically. These interventions increased tumor immunogenicity by upregulating MHC-I and improved the microenvironment by boosting CD8 + T cell infiltration, M1 macrophage polarization, monocyte differentiation, and lowering neutrophil presence [139].

KDs have been suggested to serve as effective metabolic adjuvants in cancer therapy by improving treatment response and addressing metabolic dysregulation in patients with advanced malignancies. The Keto-CARE trial (NCT03535701) investigated the impact of a well-formulated KD (WFKD) in individuals with stage IV metastatic breast cancer undergoing chemotherapy. The study demonstrated that participants were able to achieve and sustain nutritional ketosis (β-hydroxybutyrate >0.5 mM) throughout the intervention, accompanied by significant metabolic improvements [140]. Over a period of three months, the KD led to a 16% reduction in fasting plasma glucose and a 54% decrease in insulin levels (*p* < 0.01). Additionally, there was a 67% reduction in HOMA-IR scores, indicating a marked improvement in insulin sensitivity. Participants also experienced a 10% loss in body weight, primarily attributable to reductions in fat mass, as body fat percentage decreased from 45% to 37%. Nutritional ketosis was maintained for six months, facilitated by a phased approach to dietary support. The intervention was implemented in two phases to optimize adherence. In the initial three months, participants received daily ketogenic meals and intensive dietary coaching, resulting in a mean β-hydroxybutyrate level of 0.8 mM. In the subsequent three months, participants transitioned to preparing their own ketogenic meals with ongoing monitoring, successfully sustaining a mean β-hydroxybutyrate level of 0.7 mM [140].

The carbohydrate restriction (20–50 g/day) inherent in the KD effectively disrupted cancer-associated hyperglycemia while preserving lean body mass. This metabolic reprogramming may mitigate chemotherapy-induced insulin resistance and inhibit activation of the PI3K/mTOR pathway, both of which are implicated in therapeutic resistance. Importantly, the study reported no severe adverse events despite the concurrent administration of chemotherapy. The individualized dietary approach, emphasizing a variety of whole foods such as non-starchy vegetables, high-quality proteins, and satiating fats, contributed to a 75% retention rate at six months, surpassing typical adherence rates observed in dietary interventions within oncology populations [140].

Thus, this preliminary evidence proposes that structured KD protocols can safely and effectively modulate host metabolism during advanced cancer treatment. While further large-scale, controlled studies are warranted, the results of the Keto-CARE trial support the potential role of KDs as adjunctive therapies for improving metabolic parameters and enhancing treatment resilience in patients with metastatic cancer.

A preclinical performed by Talib highlighted the promising role of combining a KD with melatonin in addressing chemotherapy resistance in breast cancer. The KD exerts its effects by altering cellular metabolism, notably reducing ATP availability through a metabolic shift from glycolysis to ketolysis. This metabolic reprogramming potentially impairs the function of ATP-dependent drug efflux pumps, such as P-glycoprotein, which are commonly implicated in the development of multidrug resistance. Additionally, the KD influences tumor metabolism by lowering blood glucose levels and increasing concentrations of ketone bodies, including β-hydroxybutyrate and acetoacetate [141]. Melatonin, when administered alongside the KD, further enhances anti-tumor efficacy. It promotes apoptosis, as demonstrated by a 2.8-fold increase in caspase-3 activity compared to control groups. Moreover, melatonin downregulates key resistance-related genes, resulting in a 41% reduction in P-glycoprotein expression and a 37% decrease in glutathione S-transferase (GST) levels. The combination also significantly suppresses angiogenesis, evidenced by a 56% reduction in vascular endothelial growth factor (VEGF) expression in resistant breast cancer cell lines [141].

Experimental findings from this study show that melatonin exhibits an IC₅₀ of 8 mM in parental breast cancer cells and 12 mM in resistant lines. The resistance index (RI) in cisplatin-resistant cells was reduced from 4.2 to 1.8 following combination therapy, while vincristine-resistant cells showed a decrease in RI from 3.7 to 2.1. In vivo, the combined intervention led to a 68% reduction in tumor volume in cisplatin-resistant models and a 63% reduction in vincristine-resistant models. Notably, 70% of mice receiving the combination therapy achieved complete remission, compared to only 20% in those treated with chemotherapy alone [141]. A dual metabolic approach using the KD and melatonin may effectively overcome multidrug resistance in breast cancer. Melatonin inhibits the PI3K/AKT pathway by 34% and activates apoptosis, enhancing therapy. The 4:1 fat-to-carb KetoCal^®^ formulation offers a clinically relevant model for future trials, though further research is needed to confirm efficacy and optimize dosing in humans.

Healy et al. investigation into dietary influences on liver cancer development provided important insights into the KDKD’s potential role in modulating tumorigenesis. Although the study did not focus exclusively on KDs, the comparative evaluation of five different dietary regimens offers valuable information for understanding metabolic interventions in cancer management. The KD group exhibited the lowest tumor burden among the experimental diets, comparable to that observed with normal chow, despite the induction of obesity and glucose intolerance. Notably, tumor burden in this group showed no correlation with adiposity or fasting insulin levels, distinguishing its effects from those of sugar-rich diets. Furthermore, the KD was associated with significantly reduced hepatic triglyceride content compared to Western diets, with a 2.5-fold lower level observed in the KD group relative to the Western diet with lard [142]. Mechanistically, tumor burden positively correlated with postprandial insulin levels (*r* = 0.62, *p* < 0.01), hepatic interleukin-6 (IL-6) expression (*r* = 0.58, *p* < 0.05), and liver cholesterol accumulation (*r* = 0.71, *p* < 0.001). The KD also elicited higher hepatic expression of pro-apoptotic markers, including a 1.8-fold increase in cleaved caspase-3 compared to high-sugar diets and a 2.1-fold elevation in the tumor suppressor protein p21. Comparative analysis of dietary impacts revealed that tumor burden was substantially lower in the KD group (65 ± 18 mm³) compared to the Western diet (420 ± 95 mm³) and fructose diet (380 ± 88 mm³), with statistical significance (*p* < 0.05). Hepatic triglyceride levels were also reduced in the KD group (15 mg/g tissue) relative to the Western diet (38 mg/g tissue), while adiposity was highest in the ketogenic group (4.2 g) compared to the Western (3.8 g) and fructose (2.1 g) diets [142].

The findings suggest that dietary sugar intake, rather than fat content, serves as the primary driver of hepatocarcinogenesis in this model. The protective effects of the KD appear to be mediated through metabolic reprogramming that reduces substrate availability for lipogenesis and inflammatory pathways, enhanced apoptotic regulation via caspase-3 activation and p21-mediated cell cycle arrest, and modulation of the tumor microenvironment characterized by lower IL-6 and cholesterol-driven pro-tumor signaling. Importantly, the ketogenic formulation used (71% lard, 6% safflower oil) achieved these effects without exacerbating hepatic steatosis, distinguishing it from other high-fat diets [142]. Healy’s preclinical findings suggest carbohydrate restriction may benefit liver cancer treatment, but human trials are needed for validation. The disconnect between metabolic dysfunction and tumor suppression in KD-fed mice calls for further study of tissue-specific insulin signaling.

Zhuang et al. elucidated the synergistic relationship between metabolic interventions such as the KD and cancer therapeutics, specifically highlighting how glucose restriction potentiates metformin’s anti-tumor activity through multifaceted mechanisms. In vitro experiments revealed that low glucose conditions (0–5 mM) significantly amplified metformin’s cytotoxicity in breast (MCF7, MDAMB231, SKBR3) and ovarian (OVCAR3, PA-1) cancer cell lines. ATP depletion under low glucose, fructose, or galactose media (2.5–25 mM)—but not high glucose (25 mM)—indicated impaired glycolytic compensation as a critical factor. Furthermore, mTOR pathway inhibition, evidenced by reduced phosphorylation of AKT and S6K, occurred independently of AMPK activation, suggesting dual targeting of cancer survival pathways [143]. In vivo validation using a murine model demonstrated that a low-carbohydrate KD (93% fat, 2% carbohydrates) induced a 30% calorie restriction alongside sustained reductions in serum glucose. This dietary regimen enhanced metformin-mediated suppression of 4T1 breast tumor growth compared to standard chow, without significant weight loss in the subjects. These findings underscore the diet’s feasibility as an adjunct to pharmacotherapy [143]. The study posits a tripartite mechanistic framework for KD’s role in cancer therapy. First, glycolytic suppression reduces systemic glucose availability, limiting tumors’ access to their primary energy substrate. Second, hypoglycemia prevents metabolic compensation by cancer cells, sensitizing them to metformin-induced oxidative phosphorylation (OXPHOS) inhibition. Third, low glucose amplifies metformin’s disruption of mTOR-driven proliferative and survival signaling. Collectively, these preclinical insights suggest that KDs may counteract therapeutic resistance associated with high-glucose tumor microenvironments. However, translational validation through human trials remains essential to confirm clinical applicability. The work emphasizes the critical interplay between dietary context and metabolic drug efficacy, advocating for integrated metabolic-dietary strategies in oncology research [143].

Accumulating evidence supports KDs as versatile metabolic adjuvants in oncology, targeting cancer vulnerabilities to boost therapy. KDs act via glycolytic suppression, metabolic sensitization, and pathway modulation. Notably, KDs enhance conventional treatments, showing synergy with metformin (68% tumor suppression), chemotherapy (70% remission in resistant models), and immunotherapy (delayed progression in resistant cancers).

Overall, the Keto-CARE trial demonstrates that sustained nutritional ketosis is feasible during treatment, yielding a 16% fasting glucose reduction and a 67% decrease in insulin resistance without compromising safety. KD may reverse therapeutic resistance by downregulating PI3K/AKT signaling (34% reduction) and boosting immunogenicity via MHC-I. While preclinical data show tumor reduction and improved survival, human trials are limited. Challenges include adherence and personalized macronutrient balance. Future research should focus on randomized trials, biomarker stratification, and combining KDs with metabolic therapies like β-hydroxybutyrate to establish KD as a standard cancer treatment (Table 4).

**Table 4:** The key findings from the studies on KDs and their role in overcoming therapeutic resistance in cancer

Study focus	Key findings	Mechanism/effect	Cancer type	References
**KDs & Immune Checkpoint Blockade (ICB) Resistance**	CKD and BHB delay tumor growth in ICB-resistant prostate cancer.	MHC-I upregulation, CD8 + T cell infiltration	Prostate Cancer	[139]
	HDAC inhibition and ketogenesis enhance ICB efficacy.	M1 macrophage polarization	Prostate Cancer	[139]
**Keto-CARE Trial (Metastatic Breast Cancer)**	WFKD reduces fasting glucose (16%), insulin (54%), and HOMA-IR (67%).	Improved insulin sensitivity	Metastatic Breast Cancer	[140]
	WFKD preserves lean body mass and reduces fat mass (45% to 37%).	Metabolic reprogramming	Metastatic Breast Cancer	[140]
**KD + Melatonin in Breast Cancer**	KD reduces ATP availability, impairing drug efflux pumps (P-glycoprotein).	Metabolic shift to ketolysis	Resistant Breast Cancer	[141]
	Melatonin enhances apoptosis (2.8-fold increase in caspase-3 activity).	Downregulation of resistance genes	Resistant Breast Cancer	[141]
	Combination therapy reduces tumor volume by 68% (cisplatin-resistant models).	Angiogenesis suppression (56% VEGF reduction)	Resistant Breast Cancer	[141]
**KD in Liver Cancer**	KD reduces tumor burden (65 mm³ vs. 420 mm³ in Western diet).	Reduced IL-6, cholesterol signaling	Liver Cancer	[142]
	KD increases pro-apoptotic markers (1.8-fold caspase-3, 2.1-fold p21).	Apoptotic regulation	Liver Cancer	[142]
**KD + Metformin in Breast/Ovarian Cancer**	Low glucose amplifies metformin’s cytotoxicity (ATP depletion).	Glycolytic suppression	Breast, Ovarian Cancer	[143]
	KD enhances metformin’s mTOR pathway inhibition.	OXPHOS inhibition	Breast Cancer	[143]

## Emerging evidence from preclinical and clinical studies on kd’s anticancer potentials

### Preclinical evidence

Low-carbohydrate, high-fat KDs may prevent tumor progression and serve as supportive therapy, though limited studies have focused on colorectal cancer. Using a ketogenic infant formula (KF), researchers found that colon tumor-bearing mice fed KF exhibited preserved body and muscle mass, reduced tumor weight, and lower plasma IL-6 levels compared to controls. KF mice showed elevated blood ketone levels inversely correlated with tumor size, suggesting KF suppresses cancer progression and systemic inflammation without adverse effects on weight or muscle mass, potentially preventing cancer cachexia. This preclinical evidence supports further investigation of ketogenic interventions in colorectal cancer management [144]. KD can also exert opposite effects on tumor growth depending on a cancer’s capacity to metabolize ketone bodies. In 33 human cancer cell lines, high expression of ketolytic enzymes BDH1 and OXCT1 was associated with KD resistance, as seen in HeLa xenografts where KD accelerated growth. In contrast, PANC-1 tumors with low enzyme expression showed marked growth inhibition under KD. β-hydroxybutyrate promoted proliferation only in high-expressing cells, and enzyme downregulation sensitized resistant tumors to KD. Low ketolytic enzyme expression may serve as a predictor of KD responsiveness [145]. Notably, Maurer and colleagues demonstrated that glioma cells exhibit a metabolic disadvantage under glucose restriction, as they are unable to efficiently utilize ketone bodies for energy, unlike normal neurons. Their work combined in vitro assays (testing 3-hydroxybutyrate on rat hippocampal neurons and five glioma cell lines) with in vivo orthotopic xenograft mouse models. However, an unrestricted KD alone failed to control tumor growth, underscoring the need for combination approaches such as glycolysis inhibitors or antiangiogenic agents targeting non-oxidative pathways [146].

In a mouse model of peritoneal dissemination using colon 26 cells, a KD improved survival, health status, and anemia-related parameters compared to a regular diet, despite no significant reduction in tumor weight. These findings suggest the KD may alleviate systemic disease burden and could serve as a supportive or preventive approach against peritoneal dissemination [147]. Despite these promising findings, some preclinical models have also reported paradoxical pro-tumor effects or significant adverse outcomes, such as exacerbating metastasis and cachexia, under specific conditions, highlighting context-dependent responses [148, 149].

### Clinical evidence

In contrast to robust preclinical data, clinical investigations of KDs in oncology remain limited in scope and scale. Most human trials to date have focused on feasibility, safety, and tolerability rather than antitumor efficacy. These studies consistently demonstrate that KDs can successfully induce ketosis, moderately reduce blood glucose levels, and are generally feasible for motivated patients, often improving quality of life. Individual case reports and small observational studies suggest potential antitumor activity, proposing that KDs may exploit the Warburg effect by restricting glucose availability thereby impairing cancer cell glycolysis while promoting ketone metabolism that preferentially supports normal cells [150]. Proposed clinical benefits include mitigation of cancer cachexia, reduced muscle wasting and fatigue, lowered insulin and growth-promoting hormone levels, enhanced immune modulation, and decreased toxicity from chemotherapy and radiation [150, 151].

However, no large-scale randomized controlled trials have yet confirmed KDs as an effective standalone or adjuvant therapy in humans. While triple-negative breast cancer and other aggressive malignancies with poor prognoses underscore the urgent need for novel therapeutic strategies, current clinical evidence remains insufficient to support routine clinical adoption outside of research settings.

The KD is gaining attention as a low-cost, minimally toxic, and easily implementable nutritional intervention with biologically plausible anticancer mechanisms including metabolic targeting, anti-inflammatory effects, epigenetic modulation, and microenvironmental remodeling. Preclinical data robustly support its role in tumor suppression and synergy with conventional therapies, whereas clinical evidence is still preliminary, primarily demonstrating safety and feasibility. Patient adherence remains a key barrier to implementation. Future priorities include rigorous clinical trials to evaluate efficacy across cancer subtypes and the development of standardized combination protocols integrating KDs with established anticancer treatments.

## Challenges and controversies

The nutritional strategies demonstrate optimal potential when implemented as a complementary intervention alongside other treatment modalities. Despite limited patient sample sizes in various trials, researchers have presented compelling evidence suggesting ketogenic nutrition is safe, practical, and capable of improving outcomes for advanced cancer patients [152].

However, several significant challenges necessitate careful consideration. Dietary complexity and preparation require meticulous food measurement and a time-intensive process, making it challenging for families with demanding schedules. Additionally, the lack of a standardized protocol and composition variations may yield inconsistent results. Researchers recommend that future studies focus on developing a standardized KD treatment strategy, including precise duration and regimen, to minimize adverse effects [153].

Patient compliance challenges remain a critical limitation, as long-term adherence to rigid dietary restrictions proves difficult during cancer treatment. Younger patients may find the diet overly restrictive and unappealing. Addressing these concerns requires enhanced patient education, comprehensive support systems, and improved dietary flexibility [154].

Potential medical complications associated with KDs include frequent physiological challenges such as initial dehydration during fasting and gastrointestinal disruptions like nausea, vomiting, diarrhea, and constipation. More serious complications encompass lipid profile alterations, acute pancreatitis, cardiomyopathy, metabolic disturbances, including hyperuricemia, hyponatremia, hypoproteinemia, persistent acidosis, and hypomagnesemia, and bone and mineral metabolism issues such as hypercalciuria, hypocitraturia, kidney stone formation, and urine acidification. Recent research suggests the methodology remains tolerable as an adjuvant treatment [153].

An animal study in 2024 has raised concerns about KD potentially promoting metastasis under certain conditions. One study found that while KD reduced primary tumor growth, it also promoted tumor metastasis in the lungs of mice [148]. This highlights the complex and possibly heterogeneous tumor metabolic responses to ketones, which vary by tumor type and metabolic context.

Regarding long-term safety of the KD in cancer patients, Recent longitudinal data from a cohort of 55 advanced cancer patients followed between 2013 and 2023 provide important insights into the chronic tolerability and outcomes of KD in clinical practice. Among the 37 patients who adhered to the KD for at least three months, the median follow-up was 25 months, with a 5-year survival rate of 23.9%. Notably, patients who maintained the KD for ≥ 12 months (*n* = 21) demonstrated a markedly superior median overall survival (OS) of 55.1 months compared to 12 months in those who discontinued earlier (*n* = 32). After adjusting for background factors using inverse probability of treatment weighting, the difference remained statistically significant, suggesting a robust association between long-term dietary adherence and improved prognosis [155]. From a safety standpoint, these findings underscore that extended KD administration—up to 99 months in certain patients—appears feasible and well tolerated in a clinical oncology setting. No major diet-related toxicities necessitating discontinuation were reported, reinforcing earlier observations that KD can be safely integrated into supportive cancer care under medical supervision. Commonly monitored parameters such as renal and hepatic function, lipid profile, and body weight remained within manageable ranges, consistent with previous shorter-term studies demonstrating acceptable tolerability and metabolic stability. This long-term dataset adds to growing evidence that concerns regarding severe nutritional deficiencies or metabolic derangements under sustained KD are largely unsubstantiated when dietary protocols are properly monitored [155].

Importantly, these results also highlight that dietary adherence and patient selection critically influence outcomes. Patients who maintained KD for longer durations likely benefited from greater metabolic adaptation and improved treatment synergy, whereas early discontinuation often reflected disease progression or intolerance to dietary restrictions. Thus, structured nutritional counseling, periodic metabolic monitoring, and individualized dietary adjustments are essential for maximizing both safety and therapeutic potential [155].

Overall, the available evidence supports the long-term safety and potential survival advantage of KD as a supportive intervention in advanced cancer, particularly when maintained beyond 12 months. Nonetheless, given the observational nature of the study and potential confounding variables, randomized controlled trials are needed to confirm causality and delineate which tumor types and patient populations derive the greatest benefit. Future research should also address the mechanisms underlying prolonged metabolic adaptation, optimal macronutrient composition, and integration with standard cancer therapies to refine the clinical utility of KD in oncology.

Psychological and social implications of KD interventions present additional challenges, including extensive meal preparation requirements, limited social dining opportunities, potential feelings of isolation, variable dietary costs, and psychological stress. Recommendations for future research include a comprehensive investigation of adverse effects, ensuring safety and efficiency, and developing more adaptable dietary protocols. Despite these challenges, the ketogenic approach continues to show promise as a potential complementary cancer treatment strategy, warranting further nuanced scientific exploration.

It is a misconception to assert that the KD entirely lacks therapeutic potential in oncology, leads to nutritional deficiencies, reduces patient well-being, or adversely affects survival outcomes in individuals with cancer. Although current clinical data supporting its effectiveness remain limited, there is a clear need for future randomized trials to reinforce the evidence base and identify which patient subgroups may derive the greatest benefit [156]. A recently proposed clinical framework advocates for integrating the KD with other metabolic interventions to advance both investigational and therapeutic strategies for aggressive gliomas and other malignancies [157]. In the absence of robust, high-quality data, recommending KDs broadly to cancer patients remains contentious, and many individuals are expected to continue with conventional dietary practices. However, for those who independently opt to adopt a KD, we align with the perspective of Zemer et al. noted in their narrative review, who emphasize that “clinicians need to be informed about KD, and offer support and medical supervision for patients who self-select to follow KD. This can ensure that within the boundaries of KD, patients will make good and healthy dietary choices and prevent clinical disengagement in extreme cases” [158]. In conclusion, when properly administered, the safety profile of KDs in cancer care should no longer be a matter of dispute.

A 2020 survey by Klassen et al. involving 57 Canadian medical oncologists found that the overwhelming majority (87%) did not recommend KD to their patients [159]. This lack of endorsement stemmed from beliefs that the diet was either ineffective (31%) or had unknown clinical impacts (69%). Nearly all surveyed oncologists (96%) expressed at least one concern about KD recommendation, with predominant worries including risk of weight loss and malnutrition, potential psychological distress including anxiety and food-related fear, and possibility of additional adverse effects [159].

The ‘Prevention and Integrative Oncology’ working group within the German Cancer Society and German Society for Nutritional Medicine published a critical review expressing methodological concerns [160]. Their conclusion stated that “methodologically high-quality studies are absent, resulting in insufficient reliable evidence for the ketogenic diet. Consequently, the statement’s authors conclude that currently, employing a low-carbohydrate or ketogenic diet for this indication should be discouraged”. Interestingly, no alternative dietary approach was proposed. The statement further emphasized that KDs represent “substantial dietary limitations,” “elevate malnutrition risk within days to weeks,” and therefore result in weight reduction and deteriorating prognosis [160].

These studies reveal three dominant skeptical positions against low-carbohydrate and ketogenic diets in cancer management: (i) KDs may demonstrate no anti-tumor activity; (ii) KDs impose excessive restrictions, generating risk of psychological harm, anxiety, and food-related fear; (iii) KDs trigger weight loss, thus deteriorating patient outcomes [156].

Flexible KD protocols, including modified or less restrictive forms (such as modified Atkins diet), are preferred in resource-constrained settings to balance efficacy, cost, and cultural acceptability. Studies have confirmed ketogenic diet implementation feasibility in middle-resource countries with ongoing challenges including supplement affordability, hospital resource allocation, and ensuring continuous clinical support. In summary, socioeconomic and cultural factors significantly affect KD implementation, and tailored, flexible approaches with local community engagement, education, and consideration of food accessibility are critical for successful equity in ketogenic diet therapies, especially in cancer or epilepsy treatment in resource-limited settings. This inclusion would strengthen the review by recognizing these real-world challenges and proposing adaptive strategies to improve access and adherence beyond theoretical dietary prescriptions.

In addition, adaptive and personalized KD strategies are particularly important in translating metabolic therapies from controlled clinical environments to real-world settings with variable healthcare infrastructure. In resource-limited contexts, individualized protocols that incorporate locally available food sources, community dietitian training, and culturally appropriate meal designs can substantially enhance dietary feasibility and patient adherence. Personalization informed by metabolic profiling, genomic insights, and biomarker monitoring may further optimize dietary response, allowing clinicians to adjust macronutrient ratios and caloric targets based on individual tolerance and treatment stage. Moreover, integration of digital health technologies, such as smartphone-based tracking apps, tele-nutrition platforms, and AI-driven adherence monitoring, offers scalable tools for remote supervision, early detection of metabolic imbalances, and continuous feedback loops between patients and clinical teams. Artificial intelligence can also assist in dynamically adjusting diet plans through predictive modeling of metabolic trends and automated alerts for non-compliance or adverse effects, improving both safety and long-term adherence. These adaptive and technology-assisted frameworks hold promise for making ketogenic and related metabolic interventions both accessible and sustainable in diverse healthcare settings, narrowing disparities in nutritional oncology care across socioeconomic boundaries.

## Conclusions and future prospects

Nutritional care in oncology has advanced significantly in recent years, with greater awareness, wider implementation of clinical nutrition programs, and broader integration of multidisciplinary teams. Nevertheless, major gaps persist; nutrition specialists remain underrepresented in oncology teams, standardized malnutrition screening is still lacking, and economic as well as logistical barriers continue to limit equitable access to high-quality dietary support.

Emerging metabolic interventions such as the KD have generated substantial interest due to encouraging preclinical evidence and preliminary clinical findings. Modified, protein-sufficient KD protocols, distinct from classical epileptic versions, may target tumor cell metabolism, reduce glucose dependence, and potentially improve the efficacy of standard therapies. However, existing evidence is largely based on animal studies, pilot projects, and small non-randomized trials, while robust, large-scale RCTs remain limited. Current data mainly confirm feasibility and short-term safety, leaving questions of long-term clinical benefit unresolved.

Future progress will rely on both systemic reform and targeted research development. System-level priorities include policy initiatives to formally integrate medical nutrition into oncology care pathways, insurance coverage and reimbursement for dietitian-led interventions, routine inclusion of certified nutrition experts in cancer teams, and enhanced education programs for healthcare providers. On the research front, clearly defined priorities must guide the next phase of inquiry:

I. Establishment of standardized protocols for nutritional assessment, intervention, and outcome measurement across cancer types.
II. Stratification of patients by molecular, metabolic, and biomarker profiles to identify those most likely to benefit from KD and related dietary interventions.
III. Implementation of large, multicenter, RCTs to determine clinical efficacy, long-term safety, and optimal therapeutic combinations with standard treatments.
IV. Development of comprehensive registries and international data-sharing initiatives to promote reproducibility and guideline harmonization.

In parallel, mechanistic research should continue to elucidate the molecular and metabolic pathways modulated by nutritional interventions, with careful assessment of potential adverse effects such as enhanced metastatic potential in certain tumor contexts.

Overall, while nutritional oncology, including KD and other metabolic strategies, presents promising opportunities to improve patient outcomes and quality of life, these benefits can only be validated through coordinated, high-quality research and system-level integration. Advancing this field will require rigorous clinical investigation, biomarker-guided patient selection, and global collaboration to establish standardized, evidence-based nutritional care in oncology.

## Data Availability

No datasets were generated or analysed during the current study.

## References

[1] Arafeh R, Shibue T, Dempster JM, Hahn WC, Vazquez F. The present and future of the cancer dependency map. Nat Rev Cancer. 2025;25(1):59–73. 39468210 10.1038/s41568-024-00763-x

[2] Miller KD, Fidler-Benaoudia M, Keegan TH, Hipp HS, Jemal A, Siegel RL. Cancer statistics for adolescents and young adults, 2020. CA Cancer J Clin. 2020;70(6):443–59. 32940362 10.3322/caac.21637

[3] Damyanov C, Maslev I, Pavlov V, Avramov L. Conventional treatment of cancer realities and problems. Ann Complement Altern Med. 2018;1(1):1–9.

[4] Hardie DG. 100 years of the Warburg effect: a historical perspective. Endocr Relat Cancer. 2022;29(12):T1-13. 36094878 10.1530/ERC-22-0173

[5] Seyfried TN, Flores RE, Poff AM, D’Agostino DP. Cancer as a metabolic disease: implications for novel therapeutics. Carcinogenesis. 2014;35(3):515–27. 24343361 10.1093/carcin/bgt480 PMC3941741

[6] Seyfried TN, Shelton LM. Cancer as a metabolic disease. Nutr Metab. 2010;7:1–22. 10.1186/1743-7075-7-7 PMC2845135 20181022

[7] Plotti F, Terranova C, Luvero D, Bartolone M, Messina G, Feole L, et al. Diet and chemotherapy: the effects of fasting and ketogenic diet on cancer treatment. Chemotherapy. 2020;65(3–4):77–84. 33197913 10.1159/000510839

[8] Klement RJ. Fasting, fats, and physics: combining ketogenic and radiation therapy against cancer. Complement Med Res. 2018;25(2):102–13. 29130953 10.1159/000484045

[9] Alidadi M, Banach M, Guest PC, Bo S, Jamialahmadi T, Sahebkar A, editors. The effect of caloric restriction and fasting on cancer. Seminars in Cancer Biology; 2021: Elsevier. 10.1016/j.semcancer.2020.09.010 32977005

[10] Kalamian M. The therapeutic ketogenic diet: Harnessing glucose, insulin, and ketone metabolism. Integrative and functional medical nutrition therapy: principles and practices. Springer; 2020. pp. 335–65.

[11] Lv M, Zhu X, Wang H, Wang F, Guan W. Roles of caloric restriction, ketogenic diet and intermittent fasting during initiation, progression and metastasis of cancer in animal models: a systematic review and meta-analysis. PLoS One. 2014;9(12):e115147. 25502434 10.1371/journal.pone.0115147 PMC4263749

[12] Stafford P, Abdelwahab MG, Kim DY, Preul MC, Rho JM, Scheck AC. The ketogenic diet reverses gene expression patterns and reduces reactive oxygen species levels when used as an adjuvant therapy for glioma. Nutr Metab. 2010;7:1–11. 10.1186/1743-7075-7-74 PMC2949862 20831808

[13] Talib WH, Mahmod AI, Kamal A, Rashid HM, Alashqar AMD, Khater S, et al. Ketogenic diet in cancer prevention and therapy: molecular targets and therapeutic opportunities. Curr Issues Mol Biol. 2021;43(2):558–89. 34287243 10.3390/cimb43020042 PMC8928964

[14] Matawali A, Yeap JW, Sulaiman SF, Tan ML. The effects of ketone bodies and ketogenesis on the PI3K/AKT/mTOR signaling pathway: a systematic review. Nutr Res. 2025;139:16–49. 40381609 10.1016/j.nutres.2025.04.010

[15] Klement RJ, Fink MK. Dietary and pharmacological modification of the insulin/IGF-1 system: exploiting the full repertoire against cancer. Oncogenesis. 2016;5(2):e193. 26878387 10.1038/oncsis.2016.2 PMC5154349

[16] Kanwal R, Gupta S. Epigenetic modifications in cancer. Clin Genet. 2012;81(4):303–11. 22082348 10.1111/j.1399-0004.2011.01809.x PMC3590802

[17] Zhao Z, Shilatifard A. Epigenetic modifications of histones in cancer. Genome Biol. 2019;20:1–16. 31747960 10.1186/s13059-019-1870-5 PMC6868810

[18] Zappe K, Cichna-Markl M. Aberrant DNA methylation of ABC transporters in cancer. Cells. 2020;9(10):2281. 33066132 10.3390/cells9102281 PMC7601986

[19] Mohammad HP, Barbash O, Creasy CL. Targeting epigenetic modifications in cancer therapy: erasing the roadmap to cancer. Nat Med. 2019;25(3):403–18. 30842676 10.1038/s41591-019-0376-8

[20] Dai W, Qiao X, Fang Y, Guo R, Bai P, Liu S, et al. Epigenetics-targeted drugs: current paradigms and future challenges. Signal Transduct Target Ther. 2024;9(1):332. 39592582 10.1038/s41392-024-02039-0 PMC11627502

[21] Yoon S, Eom GH. HDAC and HDAC inhibitor: from cancer to cardiovascular diseases. Chonnam Med J. 2016;52(1):1–11. 26865995 10.4068/cmj.2016.52.1.1 PMC4742605

[22] Stephan L, Momparler R. Combination chemotherapy of cancer using the inhibitor of DNA methylation 5-aza-2’-deoxycytidine (decitabine). J Cancer Res Ther. 2015;3(5):56–65.

[23] Li J, Zhou C, Ni S, Wang S, Ni C, Yang P, Ye M. Methylated claudin-11 associated with metastasis and poor survival of colorectal cancer. Oncotarget. 2017;8(56):96249–62. 29221203 10.18632/oncotarget.21997 PMC5707097

[24] Li J, Chen C, Bi X, Zhou C, Huang T, Ni C, et al. DNA methylation of CMTM3, SSTR2, and MDFI genes in colorectal cancer. Gene. 2017;630:1–7. 28782576 10.1016/j.gene.2017.07.082

[25] Burgermeister E, Höde P, Betge J, Gutting T, Merkel A, Wu W, et al. Epigenetic silencing of tumor suppressor candidate 3 confers adverse prognosis in early colorectal cancer. Oncotarget. 2017;8(49):84714–28. 29156678 10.18632/oncotarget.20950 PMC5689568

[26] Shimizu T, LoRusso PM, Papadopoulos KP, Patnaik A, Beeram M, Smith LS, et al. Phase I first-in-human study of CUDC-101, a multitargeted inhibitor of HDACs, EGFR, and HER2 in patients with advanced solid tumors. Clin Cancer Res. 2014;20(19):5032–40. 25107918 10.1158/1078-0432.CCR-14-0570

[27] Si W, Shen J, Zheng H, Fan W. The role and mechanisms of action of MicroRNAs in cancer drug resistance. Clin Epigenetics. 2019;11:1–24. 30744689 10.1186/s13148-018-0587-8 PMC6371621

[28] Duan X, Xing Z, Qiao L, Qin S, Zhao X, Gong Y, Li X. The role of histone post-translational modifications in cancer and cancer immunity: functions, mechanisms and therapeutic implications. Front Immunol. 2024;15:1495221. 39620228 10.3389/fimmu.2024.1495221 PMC11604627

[29] Moore PC, Henderson KW, Classon M. The epigenome and the many facets of cancer drug tolerance. Adv Cancer Res. 2023;158:1–39. 36990531 10.1016/bs.acr.2022.12.002

[30] Kato S, Maeda Y, Sugiyama D, Watanabe K, Nishikawa H, Hinohara K. The cancer epigenome: non-cell autonomous player in tumor immunity. Cancer Sci. 2023;114(3):730–40. 36468774 10.1111/cas.15681 PMC9986067

[31] Lowrence RC, Subramaniapillai SG, Ulaganathan V, Nagarajan S. Tackling drug resistance with efflux pump inhibitors: from bacteria to cancerous cells. Crit Rev Microbiol. 2019;45(3):334–53. 31248314 10.1080/1040841X.2019.1607248

[32] Housman G, Byler S, Heerboth S, Lapinska K, Longacre M, Snyder N, Sarkar S. Drug resistance in cancer: an overview. Cancers. 2014;6(3):1769–92. 25198391 10.3390/cancers6031769 PMC4190567

[33] Laverdière C, Chiasson S, Costea I, Moghrabi A, Krajinovic M. Polymorphism G80A in the reduced folate carrier gene and its relationship to methotrexate plasma levels and outcome of childhood acute lymphoblastic leukemia. Blood. 2002;100(10):3832–4. 12411325 10.1182/blood.V100.10.3832

[34] Ouyang L, Shi Z, Zhao S, Wang FT, Zhou TT, Liu B, Bao JK. Programmed cell death pathways in cancer: a review of apoptosis, autophagy and programmed necrosis. Cell Prolif. 2012;45(6):487–98. 23030059 10.1111/j.1365-2184.2012.00845.x PMC6496669

[35] Green DR, Kroemer G. The pathophysiology of mitochondrial cell death. Science. 2004;305(5684):626–9. 15286356 10.1126/science.1099320

[36] Zheng JH, Viacava Follis A, Kriwacki RW, Moldoveanu T. Discoveries and controversies in BCL-2 protein‐mediated apoptosis. FEBS J. 2016;283(14):2690–700. 26411300 10.1111/febs.13527

[37] Um H-D. Bcl-2 family proteins as regulators of cancer cell invasion and metastasis: a review focusing on mitochondrial respiration and reactive oxygen species. Oncotarget. 2015;7(5):5193. 10.18632/oncotarget.6405 PMC4868680 26621844

[38] Jung J, Kim HY, Maeng J, Kim M, Shin DH, Lee K. Interaction of translationally controlled tumor protein with Apaf-1 is involved in the development of chemoresistance in HeLa cells. BMC Cancer. 2014;14:1–13. 24606760 10.1186/1471-2407-14-165 PMC4015309

[39] Obexer P, Ausserlechner MJ. X-linked inhibitor of apoptosis protein - a critical death resistance regulator and therapeutic target for personalized cancer therapy. Front Oncol. 2014;4:197. 25120954 10.3389/fonc.2014.00197 PMC4112792

[40] Pazhang Y, Jaliani HZ, Imani M, Dariushnejad H. Synergism between NF-kappa B inhibitor, celastrol, and XIAP inhibitor, embelin, in an acute myeloid leukemia cell line, HL-60. J Cancer Res Ther. 2016;12(1):155–60. 27072230 10.4103/0973-1482.150407

[41] Rigaud S, Fondanèche MC, Lambert N, Pasquier B, Mateo V, Soulas P, et al. XIAP deficiency in humans causes an X-linked lymphoproliferative syndrome. Nature. 2006;444(7115):110–4. 17080092 10.1038/nature05257

[42] Jin Z, McDonald ER, Dicker DT, El-Deiry WS. Deficient tumor necrosis factor-related apoptosis-inducing ligand (TRAIL) death receptor transport to the cell surface in human colon cancer cells selected for resistance to TRAIL-induced apoptosis. J Biol Chem. 2004;279(34):35829–39. 15155747 10.1074/jbc.M405538200

[43] Sheikh MS, Huang Y, Fernandez-Salas EA, El-Deiry WS, Friess H, Amundson S, et al. The antiapoptotic decoy receptor TRID/TRAIL-R3 is a p53-regulated DNA damage-inducible gene that is overexpressed in primary tumors of the gastrointestinal tract. Oncogene. 1999;18(28):4153–9. 10435597 10.1038/sj.onc.1202763

[44] Yaacoub K, Pedeux R, Lafite P, Jarry U, Aci-Sèche S, Bonnet P, et al. The identification of new c-FLIP inhibitors for restoring apoptosis in TRAIL-resistant cancer cells. Curr Issues Mol Biol. 2024;46(1):710–28. 38248348 10.3390/cimb46010046 PMC10814526

[45] Ambacher KK, Pitzul KB, Karajgikar M, Hamilton A, Ferguson SS, Cregan SP. The JNK- and AKT/GSK3β- signaling pathways converge to regulate Puma induction and neuronal apoptosis induced by trophic factor deprivation. PLoS One. 2012;7(10):e46885. 23056511 10.1371/journal.pone.0046885 PMC3463558

[46] Ghebeh H, Al-Khaldi S, Olabi S, Al-Dhfyan A, Al-Mohanna F, Barnawi R, et al. Fascin is involved in the chemotherapeutic resistance of breast cancer cells predominantly via the PI3K/Akt pathway. Br J Cancer. 2014;111(8):1552–61. 25117814 10.1038/bjc.2014.453 PMC4200093

[47] Wangpaichitr M, Wu C, Li YY, Nguyen DJ, Kandemir H, Shah S, et al. Exploiting ROS and metabolic differences to kill cisplatin resistant lung cancer. Oncotarget. 2017;8(30):49275. 28525376 10.18632/oncotarget.17568 PMC5564767

[48] Marullo R, Werner E, Degtyareva N, Moore B, Altavilla G, Ramalingam SS, et al. Cisplatin induces a mitochondrial-ROS response that contributes to cytotoxicity depending on mitochondrial redox status and bioenergetic functions. PLoS One. 2013;8(11):e81162. 24260552 10.1371/journal.pone.0081162 PMC3834214

[49] Poisson LM, Munkarah A, Madi H, Datta I, Hensley-Alford S, Tebbe C, et al. A metabolomic approach to identifying platinum resistance in ovarian cancer. J Ovarian Res. 2015;8:1–14. 25880539 10.1186/s13048-015-0140-8 PMC4396147

[50] Wangpaichitr M, Kandemir H, Li Y, Wu C, Nguyen D, Feun L, et al. Relationship of metabolic alterations and PD-L1 expression in cisplatin resistant lung cancer. Cell & developmental biology. 2017;6(2):183. 28819582 10.4172/2168-9296.1000183 PMC5557290

[51] Cruz-Bermúdez A, Laza-Briviesca R, Vicente-Blanco RJ, García-Grande A, Coronado MJ, Laine-Menéndez S, et al. Cisplatin resistance involves a metabolic reprogramming through ROS and PGC-1α in NSCLC which can be overcome by OXPHOS inhibition. Free Radic Biol Med. 2019;135:167–81. 30880247 10.1016/j.freeradbiomed.2019.03.009

[52] Kennedy L, Sandhu JK, Harper ME, Cuperlovic-Culf M. Role of glutathione in cancer: from mechanisms to therapies. Biomolecules. 2020;10(10):1429. 10.3390/biom10101429. 33050144 10.3390/biom10101429 PMC7600400

[53] Nunes SC, Ramos C, Lopes-Coelho F, Sequeira CO, Silva F, Gouveia-Fernandes S, et al. Cysteine allows ovarian cancer cells to adapt to hypoxia and to escape from carboplatin cytotoxicity. Sci Rep. 2018;8(1):9513. 29934500 10.1038/s41598-018-27753-y PMC6015047

[54] Li H, Stokes W, Chater E, Roy R, De Bruin E, Hu Y, et al. Decreased glutathione biosynthesis contributes to EGFR T790M-driven erlotinib resistance in non-small cell lung cancer. Cell Discovery. 2016;2(1):1–14. 10.1038/celldisc.2016.31 PMC5037574 27721983

[55] Li H, Stokes W, Chater E, Roy R, de Bruin E, Hu Y, et al. Decreased glutathione biosynthesis contributes to EGFR T790M-driven erlotinib resistance in non-small cell lung cancer. Cell Discov. 2016;2:16031. 27721983 10.1038/celldisc.2016.31 PMC5037574

[56] Zaal EA, Wu W, Jansen G, Zweegman S, Cloos J, Berkers CR. Bortezomib resistance in multiple myeloma is associated with increased serine synthesis. Cancer Metab. 2017;5:1–12. 28855983 10.1186/s40170-017-0169-9 PMC5575874

[57] Wu X, Xia J, Zhang J, Zhu Y, Wu Y, Guo J, et al. Phosphoglycerate dehydrogenase promotes proliferation and bortezomib resistance through increasing reduced glutathione synthesis in multiple myeloma. Br J Haematol. 2020;190(1):52–66. 32037523 10.1111/bjh.16503

[58] Lee MY, Yeon A, Shahid M, Cho E, Sairam V, Figlin R, et al. Reprogrammed lipid metabolism in bladder cancer with cisplatin resistance. Oncotarget. 2018;9(17):13231. 29568353 10.18632/oncotarget.24229 PMC5862574

[59] van Asten JJ, Vettukattil R, Buckle T, Rottenberg S, van Leeuwen F, Bathen TF, et al. Increased levels of choline metabolites are an early marker of docetaxel treatment response in BRCA1-mutated mouse mammary tumors: an assessment by ex vivo proton magnetic resonance spectroscopy. J Transl Med. 2015;13:1–8. 25890200 10.1186/s12967-015-0458-4 PMC4404119

[60] St-Coeur P-D, Poitras JJ, Cuperlovic-Culf M, Touaibia M, Morin PJ. Investigating a signature of Temozolomide resistance in GBM cell lines using metabolomics. J Neurooncol. 2015;125:91–102. 26311249 10.1007/s11060-015-1899-6

[61] Dar S, Chhina J, Mert I, Chitale D, Buekers T, Kaur H, et al. Bioenergetic adaptations in chemoresistant ovarian cancer cells. Sci Rep. 2017;7(1):8760. 28821788 10.1038/s41598-017-09206-0 PMC5562731

[62] You X, Jiang W, Lu W, Zhang H, Yu T, Tian J, et al. Metabolic reprogramming and redox adaptation in sorafenib-resistant leukemia cells: detected by untargeted metabolomics and stable isotope tracing analysis. Cancer Commun. 2019;39(1):17. 10.1186/s40880-019-0362-z PMC6449955 30947742

[63] Klawitter J, Kominsky DJ, Brown JL, Klawitter J, Christians U, Leibfritz D, et al. Metabolic characteristics of imatinib resistance in chronic myeloid leukaemia cells. Br J Pharmacol. 2009;158(2):588–600. 19663881 10.1111/j.1476-5381.2009.00345.x PMC2757699

[64] Li X, Lu J, Kan Q, Li X, Fan Q, Li Y, et al. Metabolic reprogramming is associated with flavopiridol resistance in prostate cancer DU145 cells. Sci Rep. 2017;7(1):5081. 28698547 10.1038/s41598-017-05086-6 PMC5506068

[65] Chen Y, León-Letelier RA, Abdel Sater AH, Vykoukal J, Dennison JB, Hanash S, et al. c-MYC-driven polyamine metabolism in ovarian cancer: from pathogenesis to early detection and therapy. Cancers (Basel). 2023;15(3):623. 10.3390/cancers15030623. 36765581 10.3390/cancers15030623 PMC9913358

[66] Zhang X, Chen Y, Hao L, Hou A, Chen X, Li Y, et al. Macrophages induce resistance to 5-fluorouracil chemotherapy in colorectal cancer through the release of putrescine. Cancer Lett. 2016;381(2):305–13. 27514455 10.1016/j.canlet.2016.08.004

[67] Cao B, Li M, Zha W, Zhao Q, Gu R, Liu L, et al. Metabolomic approach to evaluating adriamycin pharmacodynamics and resistance in breast cancer cells. Metabolomics. 2013;9:960–73. 24039617 10.1007/s11306-013-0517-x PMC3769585

[68] Sasada S, Miyata Y, Tsutani Y, Tsuyama N, Masujima T, Hihara J, Okada M. Metabolomic analysis of dynamic response and drug resistance of gastric cancer cells to 5-fluorouracil. Oncol Rep. 2012;29(3):925–31. 23232983 10.3892/or.2012.2182 PMC3597557

[69] Maria RM, Altei WF, Selistre-de-Araujo HS, Colnago LA. Impact of chemotherapy on metabolic reprogramming: characterization of the metabolic profile of breast cancer MDA-MB-231 cells using 1H HR-MAS NMR spectroscopy. J Pharm Biomed Anal. 2017;146:324–8. 28915495 10.1016/j.jpba.2017.08.038

[70] Wu W, Wang Q, Yin F, Yang Z, Zhang W, Gabra H, Li L. Identification of proteomic and metabolic signatures associated with chemoresistance of human epithelial ovarian cancer. Int J Oncol. 2016;49(4):1651–65. 27511453 10.3892/ijo.2016.3652

[71] Montopoli M, Bellanda M, Lonardoni F, Ragazzi E, Dorigo P, Froldi G, et al. Metabolic reprogramming in ovarian cancer cells resistant to cisplatin. Curr Cancer Drug Targets. 2011;11(2):226–35. 21158717 10.2174/156800911794328501

[72] Lopes-Coelho F, Gouveia-Fernandes S, Gonçalves LG, Nunes C, Faustino I, Silva F, et al. HNF1β drives glutathione (GSH) synthesis underlying intrinsic carboplatin resistance of ovarian clear cell carcinoma (OCCC). Tumor Biol. 2016;37:4813–29. 10.1007/s13277-015-4290-5 26520442

[73] Nunes SC, Lopes-Coelho F, Gouveia-Fernandes S, Ramos C, Pereira SA, Serpa J. Cysteine boosters the evolutionary adaptation to CoCl 2 mimicked hypoxia conditions, favouring carboplatin resistance in ovarian cancer. BMC Evol Biol. 2018;18:1–17. 29921232 10.1186/s12862-018-1214-1 PMC6011206

[74] Stewart DA, Winnike JH, McRitchie SL, Clark RF, Pathmasiri WW, Sumner SJ. Metabolomics analysis of hormone-responsive and triple-negative breast cancer cell responses to Paclitaxel identify key metabolic differences. J Proteome Res. 2016;15(9):3225–40. 27447733 10.1021/acs.jproteome.6b00430 PMC5034715

[75] Lee S, Jang WJ, Choi B, Joo SH, Jeong CH. Comparative metabolomic analysis of HPAC cells following the acquisition of erlotinib resistance. Oncol Lett. 2017;13(5):3437–44. 28529573 10.3892/ol.2017.5940 PMC5431587

[76] Dewar BJ, Keshari K, Jeffries R, Dzeja P, Graves LM, Macdonald JM. Metabolic assessment of a novel chronic myelogenous leukemic cell line and an Imatinib resistant subline by 1 H NMR spectroscopy. Metabolomics. 2010;6:439–50. 20676217 10.1007/s11306-010-0204-0 PMC2899017

[77] You X, Jiang W, Lu W, Zhang H, Yu T, Tian J, et al. Metabolic reprogramming and redox adaptation in sorafenib-resistant leukemia cells: detected by untargeted metabolomics and stable isotope tracing analysis. Cancer Commun. 2019;39:1–13. 10.1186/s40880-019-0362-z PMC6449955 30947742

[78] Kominsky DJ, Klawitter J, Brown JL, Boros LG, Melo JV, Eckhardt SG, Serkova NJ. Abnormalities in glucose uptake and metabolism in imatinib-resistant human BCR-ABL–positive cells. Clin Cancer Res. 2009;15(10):3442–50. 19401345 10.1158/1078-0432.CCR-08-3291

[79] Van Dang C. Cancer metabolism: The known, unknowns. 2018. p. 1. 10.1016/j.bbcan.2018.07.006 30336841

[80] DeBerardinis RJ, Chandel NS. Fundamentals of cancer metabolism. Sci Adv. 2016;2(5):e1600200. 27386546 10.1126/sciadv.1600200 PMC4928883

[81] Muñoz-Pinedo C, El Mjiyad N, Ricci J-E. Cancer metabolism: current perspectives and future directions. Cell Death Dis. 2012;3(1):e248-e. 22237205 10.1038/cddis.2011.123 PMC3270265

[82] Kato Y, Maeda T, Suzuki A, Baba Y. Cancer metabolism: new insights into classic characteristics. Jpn Dent Sci Rev. 2018;54(1):8–21. 29628997 10.1016/j.jdsr.2017.08.003 PMC5884251

[83] Ghaffari P, Mardinoglu A, Nielsen J. Cancer metabolism: a modeling perspective. Front Physiol. 2015;6:382. 26733270 10.3389/fphys.2015.00382 PMC4679931

[84] Frezza C. Metabolism and cancer: the future is now. Br J Cancer. 2020;122(2):133–5. 31819199 10.1038/s41416-019-0667-3 PMC6978144

[85] Li T, Le A. Glutamine metabolism in cancer. Heterogeneity Cancer Metabolism. 2018;1063:13–32. 10.1007/978-3-319-77736-8_2 29946773

[86] Cluntun AA, Lukey MJ, Cerione RA, Locasale JW. Glutamine metabolism in cancer: understanding the heterogeneity. Trends Cancer. 2017;3(3):169–80. 28393116 10.1016/j.trecan.2017.01.005 PMC5383348

[87] Lyu H, Bao S, Cai L, Wang M, Liu Y, Sun Y, et al. The role and research progress of serine metabolism in tumor cells. Front Oncol. 2025;15:1509662. 40265021 10.3389/fonc.2025.1509662 PMC12011608

[88] Yang M, Vousden KH. Serine and one-carbon metabolism in cancer. Nat Rev Cancer. 2016;16(10):650–62. 27634448 10.1038/nrc.2016.81

[89] Reina-Campos M, Diaz-Meco MT, Moscat J. The complexity of the serine glycine one-carbon pathway in cancer. J Cell Biol. 2019;219(1):e201907022. 10.1083/jcb.201907022 PMC7039202 31690618

[90] Li F, Yin Y, Tan B, Kong X, Wu G. Leucine nutrition in animals and humans: mTOR signaling and beyond. Amino Acids. 2011;41:1185–93. 21773813 10.1007/s00726-011-0983-2

[91] Katsanos CS, Kobayashi H, Sheffield-Moore M, Aarsland A, Wolfe RR. A high proportion of leucine is required for optimal stimulation of the rate of muscle protein synthesis by essential amino acids in the elderly. Am J Physiol Endocrinol Metab. 2006. 10.1152/ajpendo.00488.2005. 16507602 10.1152/ajpendo.00488.2005

[92] Vianna D, Teodoro GFR, Torres-Leal FL, Tirapegui J. Protein synthesis regulation by leucine. Braz J Pharm Sci. 2010;46:29–36.

[93] Sheen J-H, Zoncu R, Kim D, Sabatini DM. Defective regulation of autophagy upon leucine deprivation reveals a targetable liability of human melanoma cells *in vitro* and *in vivo*. Cancer Cell. 2011;19(5):613–28. 21575862 10.1016/j.ccr.2011.03.012 PMC3115736

[94] Mayers JR, Wu C, Clish CB, Kraft P, Torrence ME, Fiske BP, et al. Elevation of circulating branched-chain amino acids is an early event in human pancreatic adenocarcinoma development. Nat Med. 2014;20(10):1193–8. 25261994 10.1038/nm.3686 PMC4191991

[95] Viana LR, Tobar N, Busanello ENB, Marques AC, de Oliveira AG, Lima TI, et al. Leucine-rich diet induces a shift in tumour metabolism from glycolytic towards oxidative phosphorylation, reducing glucose consumption and metastasis in Walker-256 tumour-bearing rats. Sci Rep. 2019;9(1):15529. 31664147 10.1038/s41598-019-52112-w PMC6820796

[96] Xiao F, Wang C, Yin H, Yu J, Chen S, Fang J, Guo F. Leucine deprivation inhibits proliferation and induces apoptosis of human breast cancer cells via fatty acid synthase. Oncotarget. 2016;7(39):63679. 27579768 10.18632/oncotarget.11626 PMC5325395

[97] Storck LJ, Ruehlin M, Gaeumann S, Gisi D, Schmocker M, Meffert PJ, et al. Effect of a leucine-rich supplement in combination with nutrition and physical exercise in advanced cancer patients: a randomized controlled intervention trial. Clin Nutr. 2020;39(12):3637–44. 32340904 10.1016/j.clnu.2020.04.008

[98] Zhang M, Zhang Q, Huang S, Lu Y, Peng M. Impact of ketogenic diets on cancer patient outcomes: a systematic review and meta-analysis. Front Nutr. 2025;12:1535921. 40756563 10.3389/fnut.2025.1535921 PMC12313497

[99] Wallis J. The Ketogenic Diet for Cancer Patients: A Narrative Review. 2018.

[100] Cohen CW, Fontaine KR, Arend RC, Gower BA. A ketogenic diet is acceptable in women with ovarian and endometrial cancer and has no adverse effects on blood lipids: a randomized, controlled trial. Nutr Cancer. 2020;72(4):584–94. 31352797 10.1080/01635581.2019.1645864

[101] Kämmerer U, Klement RJ, Joos FT, Sütterlin M, Reuss-Borst M. Low carb and ketogenic diets increase quality of life, physical performance, body composition, and metabolic health of women with breast cancer. Nutrients. 2021;13(3):1029. 10.3390/nu13031029. 33806775 10.3390/nu13031029 PMC8004887

[102] Kang CM, Yun B, Kim M, Song M, Kim YH, Lee SH, et al. Postoperative serum metabolites of patients on a low carbohydrate ketogenic diet after pancreatectomy for pancreatobiliary cancer: a nontargeted metabolomics pilot study. Sci Rep. 2019;9(1):16820. 31727967 10.1038/s41598-019-53287-y PMC6856065

[103] Khodabakhshi A, Akbari ME, Mirzaei HR, Seyfried TN, Kalamian M, Davoodi SH. Effects of ketogenic metabolic therapy on patients with breast cancer: a randomized controlled clinical trial. Clin Nutr. 2021;40(3):751–8. 32703721 10.1016/j.clnu.2020.06.028

[104] Ok JH, Lee H, Chung HY, Lee SH, Choi EJ, Kang CM, Lee SM. The potential use of a ketogenic diet in pancreatobiliary cancer patients after pancreatectomy. Anticancer Res. 2018;38(11):6519–27. 30396981 10.21873/anticanres.13017

[105] Lu Y, Yang Y-Y, Zhou M-W, Liu N, Xing H-Y, Liu X-X, Li F. Ketogenic diet attenuates oxidative stress and inflammation after spinal cord injury by activating Nrf2 and suppressing the NF-κB signaling pathways. Neurosci Lett. 2018;683:13–8. 29894768 10.1016/j.neulet.2018.06.016

[106] Osawa Y, Nagaki M, Banno Y, Brenner DA, Asano T, Nozawa Y, et al. <article-title update="added">Tumor necrosis factor alpha-induced interleukin-8 production via NF-κB and phosphatidylinositol 3-kinase/Akt pathways inhibits cell apoptosis in human hepatocytes. Infect Immun. 2002;70(11):6294–301. 12379708 10.1128/IAI.70.11.6294-6301.2002 PMC130316

[107] Yang C, Sudderth J, Dang T, Bachoo RG, McDonald JG, DeBerardinis RJ. Glioblastoma cells require glutamate dehydrogenase to survive impairments of glucose metabolism or Akt signaling. Cancer Res. 2009;69(20):7986–93. 19826036 10.1158/0008-5472.CAN-09-2266 PMC2764330

[108] Kassab AE. Recent advances in targeting COX-2 for cancer therapy: a review. RSC Med Chem. 202516:2974-3002 10.1039/d5md00196j PMC12082340 40386345

[109] Jin K, Qian C, Lin J, Liu B. Cyclooxygenase-2-prostaglandin E2 pathway: a key player in tumor-associated immune cells. Front Oncol. 2023;13:1099811. 36776289 10.3389/fonc.2023.1099811 PMC9911818

[110] Laronha H, Carpinteiro I, Portugal J, Azul A, Polido M, Petrova KT, et al. Challenges in matrix metalloproteinases inhibition. Biomolecules. 2020;10(5):717. 32380782 10.3390/biom10050717 PMC7277161

[111] Said AH, Raufman J-P, Xie G. The role of matrix metalloproteinases in colorectal cancer. Cancers. 2014;6(1):366–75. 24518611 10.3390/cancers6010366 PMC3980606

[112] Mondal S, Adhikari N, Banerjee S, Amin SA, Jha T. Matrix metalloproteinase-9 (MMP-9) and its inhibitors in cancer: a minireview. Eur J Med Chem. 2020;194:112260. 32224379 10.1016/j.ejmech.2020.112260

[113] Zhang N, Liu C, Jin L, Zhang R, Wang T, Wang Q, et al. Ketogenic diet elicits antitumor properties through inducing oxidative stress, inhibiting MMP-9 expression, and rebalancing M1/M2 tumor-associated macrophage phenotype in a mouse model of colon cancer. J Agric Food Chem. 2020;68(40):11182–96. 32786841 10.1021/acs.jafc.0c04041

[114] Zhang H, Shang YP, Chen Hy, Li J. Histone deacetylases function as novel potential therapeutic targets for cancer. Hepatol Res. 2017;47(2):149–59. 27457249 10.1111/hepr.12757

[115] Sabari BR, Zhang D, Allis CD, Zhao Y. Metabolic regulation of gene expression through histone acylations. Nat Rev Mol Cell Biol. 2017;18(2):90–101. 27924077 10.1038/nrm.2016.140 PMC5320945

[116] West AC, Johnstone RW. New and emerging HDAC inhibitors for cancer treatment. J Clin Invest. 2014;124(1):30–9. 24382387 10.1172/JCI69738 PMC3871231

[117] Liu K, Li F, Sun Q, Lin N, Han H, You K, et al. P53 β-hydroxybutyrylation attenuates p53 activity. Cell Death Dis. 2019;10(3):243. 30858356 10.1038/s41419-019-1463-y PMC6411878

[118] Motoshima H, Goldstein BJ, Igata M, Araki E. AMPK and cell proliferation–AMPK as a therapeutic target for atherosclerosis and cancer. J Physiol. 2006;574(1):63–71. 16613876 10.1113/jphysiol.2006.108324 PMC1817805

[119] Wang Z, Wang N, Liu P, Xie X. AMPK and cancer. AMP-activated Protein Kinase. 2016;203:26. 10.1007/978-3-319-43589-3_9 27812982

[120] Lee Y-K, Park SY, Kim Y-M, Lee WS, Park OJ. AMP kinase/cyclooxygenase-2 pathway regulates proliferation and apoptosis of cancer cells treated with Quercetin. Exp Mol Med. 2009;41(3):201–7. 19293639 10.3858/emm.2009.41.3.023 PMC2679247

[121] Umezawa S, Higurashi T, Nakajima A. AMPK: therapeutic target for diabetes and cancer prevention. Curr Pharm Des. 2017;23(25):3629–44. 28714409 10.2174/0929867324666170713150440

[122] Bose S, Allen AE, Locasale JW. The molecular link from diet to cancer cell metabolism. Mol Cell. 2020;78(6):1034–44. 32504556 10.1016/j.molcel.2020.05.018 PMC7305994

[123] Pekarek L, Torres-Carranza D, Fraile-Martinez O, García-Montero C, Pekarek T, Saez MA, et al. An overview of the role of MicroRNAs on carcinogenesis: a focus on cell cycle, angiogenesis and metastasis. Int J Mol Sci. 2023;24(8):7268. 10.3390/ijms24087268. 37108432 10.3390/ijms24087268 PMC10139430

[124] Loboda A, Sobczak M, Jozkowicz A, Dulak J. TGF-β1/Smads and miR‐21 in renal fibrosis and inflammation. Mediators Inflamm. 2016;2016(1):8319283. 27610006 10.1155/2016/8319283 PMC5005604

[125] Chang K, Miller N, Kheirelseid E, Ingoldsby H, Hennessy E, Curran C, et al. MicroRNA-21 and PDCD4 expression in colorectal cancer. European Journal of Surgical Oncology (EJSO). 2011;37(7):597–603. 21546206 10.1016/j.ejso.2011.04.001

[126] Arndt GM, Dossey L, Cullen LM, Lai A, Druker R, Eisbacher M, et al. Characterization of global MicroRNA expression reveals oncogenic potential of miR-145 in metastatic colorectal cancer. BMC Cancer. 2009;9:1–17. 19843336 10.1186/1471-2407-9-374 PMC2770572

[127] King C, Wang L, Winograd R, Madison B, Mongroo P, Johnstone C, Rustgi AK. LIN28B fosters colon cancer migration, invasion and transformation through let-7-dependent and-independent mechanisms. Oncogene. 2011;30(40):4185–93. 21625210 10.1038/onc.2011.131 PMC3165068

[128] Sha D, Lee AM, Shi Q, Alberts SR, Sargent DJ, Sinicrope FA, et al. Association study of the let-7 miRNA-complementary site variant in the 3′ untranslated region of the KRAS gene in stage III colon cancer (NCCTG N0147 clinical trial). Clin Cancer Res. 2014;20(12):3319–27. 24727325 10.1158/1078-0432.CCR-14-0069 PMC4084689

[129] Oue N, Anami K, Schetter AJ, Moehler M, Okayama H, Khan MA, et al. High miR-21 expression from FFPE tissues is associated with poor survival and response to adjuvant chemotherapy in colon cancer. Int J Cancer. 2014;134(8):1926–34. 24122631 10.1002/ijc.28522 PMC3947446

[130] Mukherjee P, Augur ZM, Li M, Hill C, Greenwood B, Domin MA, et al. Therapeutic benefit of combining calorie-restricted ketogenic diet and glutamine targeting in late-stage experimental glioblastoma. Commun Biology. 2019;2(1):200. 10.1038/s42003-019-0455-x PMC6541653 31149644

[131] Martínez-Reza I, Díaz L, García-Becerra R. Preclinical and clinical aspects of TNF-α and its receptors TNFR1 and TNFR2 in breast cancer. J Biomed Sci. 2017;24:1–8. 29202842 10.1186/s12929-017-0398-9 PMC5713022

[132] Hwang CY, Choe W, Yoon KS, Ha J, Kim SS, Yeo EJ, et al. Molecular mechanisms for ketone body metabolism, signaling functions, and therapeutic potential in cancer. Nutrients. 2022;14(22):4932. 10.3390/nu14224932. 36432618 10.3390/nu14224932 PMC9694619

[133] Bandera-Merchan B, Boughanem H, Crujeiras AB, Macias-Gonzalez M, Tinahones FJ. Ketotherapy as an epigenetic modifier in cancer. Rev Endocr Metab Disord. 2020;21:509–19. 32514818 10.1007/s11154-020-09567-4

[134] Ruan H-B, Crawford PA. Ketone bodies as epigenetic modifiers. Curr Opin Clin Nutr Metab Care. 2018;21(4):260–6. 29697540 10.1097/MCO.0000000000000475

[135] Hursting SD, Ford NA, Dunlap SM, Hursting MJ, Lashinger LM. Calorie restriction and cancer prevention: established and emerging mechanisms. In: Obesity, Inflammation and Cancer. Springer; 2013. p. 363–79.

[136] Duan Y, Zeng L, Zheng C, Song B, Li F, Kong X, Xu K. Inflammatory links between high fat diets and diseases. Front Immunol. 2018;9:2649. 30483273 10.3389/fimmu.2018.02649 PMC6243058

[137] Parida S, Siddharth S, Sharma D. Adiponectin, obesity, and cancer: clash of the bigwigs in health and disease. Int J Mol Sci. 2019;20(10):2519. 31121868 10.3390/ijms20102519 PMC6566909

[138] Li J, Zhang H, Dai Z. Cancer treatment with the ketogenic diet: a systematic review and meta-analysis of animal studies. Front Nutr. 2021;8:594408. 34179051 10.3389/fnut.2021.594408 PMC8219874

[139] Murphy S, Rahmy S, Gan D, Liu G, Zhu Y, Manyak M, et al. Ketogenic diet alters the epigenetic and immune landscape of prostate cancer to overcome resistance to immune checkpoint blockade therapy. Cancer Res. 2024;84(10):1597–612. 38588411 10.1158/0008-5472.CAN-23-2742 PMC11096030

[140] Buga A, Harper DG, Sapper TN, Hyde PN, Fell B, Dickerson R, et al. Feasibility and metabolic outcomes of a well-formulated ketogenic diet as an adjuvant therapeutic intervention for women with stage IV metastatic breast cancer: the Keto-CARE trial. PLoS One. 2024;19(1):e0296523. 38166036 10.1371/journal.pone.0296523 PMC10760925

[141] Talib WH. A ketogenic diet combined with melatonin overcomes cisplatin and vincristine drug resistance in breast carcinoma syngraft. Nutrition. 2020;72:110659. 31986320 10.1016/j.nut.2019.110659

[142] Healy ME, Chow JD, Byrne FL, Breen DS, Leitinger N, Li C, et al. Dietary effects on liver tumor burden in mice treated with the hepatocellular carcinogen diethylnitrosamine. J Hepatol. 2015;62(3):599–606. 25450719 10.1016/j.jhep.2014.10.024 PMC4336610

[143] Zhuang Y, Chan DK, Haugrud AB, Miskimins WK. Mechanisms by which low glucose enhances the cytotoxicity of Metformin to cancer cells both in vitro and in vivo. PLoS One. 2014;9(9):e108444. 25254953 10.1371/journal.pone.0108444 PMC4177919

[144] Nakamura K, Tonouchi H, Sasayama A, Ashida K. A ketogenic formula prevents tumor progression and cancer cachexia by attenuating systemic inflammation in colon 26 tumor-bearing mice. Nutrients. 2018;10(2):206. 29443873 10.3390/nu10020206 PMC5852782

[145] Zhang J, Jia P-P, Liu Q-L, Cong M-H, Gao Y, Shi H-P, et al. Low ketolytic enzyme levels in tumors predict ketogenic diet responses in cancer cell lines in vitro and in vivo. J Lipid Res. 2018;59(4):625–34. 29414764 10.1194/jlr.M082040 PMC5880499

[146] Maurer GD, Brucker DP, Bähr O, Harter PN, Hattingen E, Walenta S, et al. Differential utilization of ketone bodies by neurons and glioma cell lines: a rationale for ketogenic diet as experimental glioma therapy. BMC Cancer. 2011;11:315. 21791085 10.1186/1471-2407-11-315 PMC3199865

[147] Kasumi E, Sato N. A ketogenic diet improves the prognosis in a mouse model of peritoneal dissemination without tumor regression. J Clin Biochem Nutr. 2019;64(3):201–8. 31138953 10.3164/jcbn.18-103 PMC6529699

[148] Su Z, Liu Y, Xia Z, Rustgi AK, Gu W. An unexpected role for the ketogenic diet in triggering tumor metastasis by modulating BACH1-mediated transcription. Sci Adv. 2024;10(23):eadm9481. 38838145 10.1126/sciadv.adm9481 PMC11152127

[149] Ferrer M, Mourikis N, Davidson EE, Kleeman SO, Zaccaria M, Habel J et al. Ketogenic diet promotes tumor ferroptosis but induces relative corticosterone deficiency that accelerates cachexia. Cell Metab. 2023;35(7):1147-62.e7. 10.1016/j.cmet.2023.05.008 PMC11037504 37311455

[150] Tran Q, Lee H, Kim C, Kong G, Gong N, Kwon SH, et al. Revisiting the Warburg Effect: diet-based strategies for cancer prevention. Biomed Res Int. 2020;2020:8105735. 32802877 10.1155/2020/8105735 PMC7426758

[151] Ludwig DS. The ketogenic diet: evidence for optimism but High-Quality research needed. J Nutr. 2020;150(6):1354–9. 31825066 10.1093/jn/nxz308 PMC7269727

[152] Deng Q, Lv R, Zou T. The effects of the ketogenic diet on cancer treatment: a narrative review. Eur J Cancer Prev. 2025;34(4):291–300. 39365252 10.1097/CEJ.0000000000000918

[153] Batch JT, Lamsal SP, Adkins M, Sultan S, Ramirez MN. Advantages and disadvantages of the ketogenic diet: a review article. Cureus. 2020;12(8):e9639. 32923239 10.7759/cureus.9639 PMC7480775

[154] Weber DD, Aminzadeh-Gohari S, Tulipan J, Catalano L, Feichtinger RG, Kofler B. Ketogenic diet in the treatment of cancer - where do we stand? Mol Metab. 2020;33:102–21. 31399389 10.1016/j.molmet.2019.06.026 PMC7056920

[155] Egashira R, Matsunaga M, Miyake A, Hotta S, Nagai N, Yamaguchi C, et al. Long-term effects of a ketogenic diet for cancer. Nutrients. 2023;15(10):2334. 10.3390/nu15102334. 37242217 10.3390/nu15102334 PMC10221628

[156] Klement RJ. Is the ketogenic diet still controversial in cancer treatment? Expert Rev Anticancer Ther. 2025;25(9):993–7. 40530973 10.1080/14737140.2025.2522936

[157] Duraj T, Kalamian M, Zuccoli G, Maroon JC, D’Agostino DP, Scheck AC, et al. Clinical research framework proposal for ketogenic metabolic therapy in glioblastoma. BMC Med. 2024;22(1):578. 39639257 10.1186/s12916-024-03775-4 PMC11622503

[158] Zemer A, Samaei S, Yoel U, Biderman A, Pincu Y. Ketogenic diet in clinical populations—a narrative review. Front Med (Lausanne). 2024;11:1432717. 39534224 10.3389/fmed.2024.1432717 PMC11554467

[159] Klassen PN, Goldenberg BA, Lambert P, Vagianos K, Kim CA. Ketogenic and low-sugar diets for patients with cancer: perceptions and practices of medical oncologists in Canada. Support Care Cancer. 2020;28(11):5243–9. 32090285 10.1007/s00520-020-05361-9

[160] Schmidt L, Mathies V, von Grundherr J, Rubin D, Hübner J, for the Working Group Prevention and Integrative Oncology in the German Cancer Society and the German Society for Nutritional Medicine. Ketogenic and low-carbohydrate diets in people with cancer. A statement by the working group on prevention and integrative oncology (PRIO) in the German cancer society (GCS) and the German society for nutritional medicine (DGEM). Ernahrungsumschau. 2022;69(7):106–11.

